# Indigenous communities and influenza: protocol for a systematic review and meta-analysis

**DOI:** 10.1186/s13643-023-02319-w

**Published:** 2023-08-30

**Authors:** D. E. Alves, O. Rogeberg, L. Sattenspiel, S. Mamelund

**Affiliations:** 1 Work Research Institute and Centre for Research on Pandemics and Society, OsloMet — Oslo Metropolitan University, Oslo, Norway; 2 Frisch Center, University of Oslo, Oslo, Norway; 3 Department of Anthropology, University of Missouri, Columbia, MO USA; 4 Centre for Research on Pandemics and Society, OsloMet — Oslo Metropolitan University, Oslo, Norway

**Keywords:** Protocol, Meta-analysis, Systematic review, Indigenous, Influenza, Pandemic

## Abstract

**Background:**

Several studies have documented that specific Indigenous groups have been disproportionately affected by previous pandemics. The objective of this paper is to describe the protocol to be used in a review and meta-analysis of the literature on Indigenous groups and influenza. Using this protocol as a guide, a future study will provide a comprehensive historical overview of pre-COVID impact of influenza on Indigenous groups by combining data from the last five influenza pandemics and seasonal influenza up to date.

**Methods/principle findings:**

The review will include peer-reviewed original studies published in English, Spanish, Portuguese, Swedish, Danish, and Norwegian. Records will be identified through systematic literature search in eight databases: Embase, MEDLINE, CINAHL, Web of Science, Academic Search Ultimate, SocINDEX, ASSIA, and Google Scholar. Results will be summarized narratively and using meta-analytic strategies.

**Discussion:**

To our knowledge, there is no systematic review combining historical data on the impact of both seasonal and pandemic influenza on Indigenous populations. By summarizing results within and across Indigenous groups, different countries, and historical periods, as well as research in six different languages, we aim to provide information on how strong the risk for influenza is among Indigenous groups and how consistent this risk is across groups, regions, time, and seasonal versus the specific pandemic influenza strains.

**Systematic review registration:**

PROSPERO CRD42021246391

**Supplementary Information:**

The online version contains supplementary material available at 10.1186/s13643-023-02319-w.

## Introduction

In 2020, Indigenous people comprised 6% of the world’s population with an estimated 476 million people [1]. History has shown that Indigenous groups have been disproportionately affected by influenza and more likely to experience severe outcomes compared to non-Indigenous counterparts [2].

Influenza remains one of the world’s greatest public health challenges with an estimated 1 billion cases of seasonal influenza every year. About 3–5 million of these cases are severe, leading to 290,000–650,000 deaths globally [3, 4]. The 2009 “swine flu” pandemic resulted in an estimated 60 million cases, 274,304 hospitalizations, and 12,469 deaths in the USA alone [5]. Such morbidity and mortality figures result in a huge economic burden due to direct healthcare spending and indirect costs, such as loss of productivity as a consequence of workplace absenteeism stemming from employees’ own sickness or care for others [6–9]. One-hundred years ago, the “Spanish flu” pandemic of 1918–1920 took the lives of ~50 million people globally (2.5%). Influenza pandemics have appeared 3–4 times each century since the first documented pandemic in 1510 [10]. Based on earlier pandemics, the death toll of the next pandemic could range from rather mild (as in 2009) to devasting (as in 1918). A severe influenza pandemic akin to that of 1918 could lead to 80 million deaths and cost as much as 5% of the global GDP [11]. Subsequent, less severe pandemics occurred in 1957–1958, 1968–1969, and 2009–2010, still resulting in 100,000–400,000 global deaths [12].

Multiple studies report raised risks of adverse medical outcomes for Indigenous groups during influenza pandemics. For example, during the 1918 influenza pandemic, the Indigenous Māori on New Zealand and the Sami population in Norway had respectively 4–6 and 7 times higher mortality risks than non-Indigenous counterparts [13, 14]. These risk disparities appear persistent across time and space. In the 2009 pandemic, Indigenous groups in North America, Oceania, and the Pacific had 3–8 times higher pandemic mortality than the majority populations [15, 16]. Studies of Indigenous populations in North America and Oceania find raised risk for adverse health outcomes both today and 100 years ago [15–24].

The reasons for these ethnic disparities are complex and poorly understood, and they have been an area of limited research. Genetic, epidemiological, and social science research is also carried out in silos and does not incorporate adequate communication among academic disciplines and, more importantly, between researchers and the Indigenous communities they are researching. Influenza research on Indigenous groups, including research on influenza preparedness planning, often focuses on specific areas such as North America [25] or Oceania [26], adding to the fragmented silo understanding.

Influenza-related ethnic disparities in the 2009 influenza pandemic may be explained in part by a higher prevalence of risk factors for severe influenza outcomes in Indigenous communities (e.g., 2–7 times higher risk of diabetes mellitus, obesity, asthma, or chronic obstructive pulmonary disease and a greater number of pregnancies at young age). Less documented factors include those associated with the risk of infection (e.g., crowding, family size, and poverty), differences in access to health care, and a greater genetic susceptibility [18]. Finally, the fact that many Indigenous populations have historically lived in more isolated areas means that their opportunities for immunological imprinting and their lifetime experience with influenza are different from those of non-isolated populations, which almost certainly affects the risk for and severity of influenza pandemics that reach them. Isolation may also be a manufactured vulnerability since many Indigenous communities were dispossessed from their homes during times of colonization [27]. Indigenous populations in the USA, Canada [27], and Australia [28, 29] are prioritized for both seasonal and pandemic influenza vaccines but tend to have lower vaccination rates than non-Indigenous populations [18, 30, 31]. Thus, lower vaccination rates in Indigenous, compared to non-Indigenous communities, may also explain influenza disparities.

Although outside the scope of this research, there is support that this raised influenza vulnerability in Indigenous communities also appear for pandemics not involving influenza, such as COVID-19 [32–34]. Nevertheless, there are also studies showing specific countries and historical periods in which there was limited disproportionate morbidity or mortality in Indigenous communities, compared to non-Indigenous ones [34]. Such cases may be particularly important to identify, in that they may indicate the presence or absence of factors influencing the risk disparities faced by Indigenous groups that can be utilized in pandemic planning to reduce these disparities.

In this study, we present a protocol for the first systematic review and meta-analysis on the association between Indigenous background and influenza. The protocol provides a structured and rigorous method that will allow us to identify appropriate articles with reduced bias towards the eventual outcomes of the review. In this research, we focus on both seasonal influenza and the last five pre-COVID-19 influenza pandemics (1889–1990, 1918–1920, 1957–1958, 1969–1970, 2009–2010) with the following review question: How strong is the association between Indigenous background and influenza outcomes, and is the association strong enough to be important for incorporated into prevention policies above and beyond the role of medical risk factors?

## Methods

This protocol is in line with the reporting guidance provided in the Preferred Reporting Items for Systematic Reviews and Meta-Analyses Protocols statement [35] (see checklist Additional file 1). In addition, the protocol has been registered within the International Prospective Register of Systematic Reviews (PROSPERO) database (registration number CRD42021246391) [36]. Our methodology is a protocol-driven comprehensive review and synthesis of quantitative data focusing on the review question presented in the previous paragraph. Influenza is defined as quantifiable outcomes (infection, hospitalization, or mortality), and pandemic influenza refers to the last five influenza pandemics (1889, 1918, 1957, 1968, 2009). Indigenous is defined as “initial occupants and descendants of a nation who have experienced colonization and allocation to minority status” [37].

### Systematic literature search

Initial searches in a preliminary project were conducted during January and February 2021 in eight databases: Embase, MEDLINE, CINAHL, Web of Science, Academic Search Ultimate, SocINDEX, ASSIA, and Google Scholar. The search strategy was developed and piloted in collaboration with research librarians to combine sensitivity and precision. Searches were conducted according to PICO criteria. PICO’s acronym means Population, Intervention (or Exposure), Comparison and Outcome [38]. The intervention was broadened to also include Exposure, which in the case of our review was “influenza”. Thus, the key PICO terms in our study were as follows: Indigenous (P), influenza (I), healthy/well (C), and infection/hospitalization/death (O). The result of the piloted tests indicated that the most optimal search, in terms of specificity and sensitivity, was that the final search strategy comprised of two of the four criteria in the PICO acronym: Indigenous (P for Population) and influenza (I for Intervention or Exposure). These terms with synonyms and related terms were combined within each database (see Additional file 2 for search strategy). No language nor publication data restrictions were applied at this stage. Two reviewers screened the abstracts in this preliminary project, and included full-text studies were transferred to the software Covidence [39]. Upon completion of screening of these studies in Covidence, by Fall 2023, we will screen an expanded set of studies that includes the articles in the reference lists of included studies and relevant reviews on the topic. This will allow us to identify additional eligible studies not retrieved by our search.

### Eligibility criteria

This protocol covers seasonal influenza up to the literature search date and the last five pre-COVID-19 influenza pandemics, which occurred during the years 1889–1990, 1918–1920, 1957–1958, 1969–1970, and 2009–2010. We include quantitative studies statistically investigating the relationship between having an Indigenous background (compared to non-Indigenous) and disease outcome (morbidity, hospitalization, and mortality). This relationship between Indigenous background and influenza must also be empirically investigated and reported in full-text articles in peer-reviewed journals. Indigenous background is captured by key words such as Native, Aboriginal, and Sami (see Additional file 2 for more examples). Because we wish to collect information from other periods and times, as well as across disciplines with different connotations regarding post-colonialism, some of the words included in the search are considered derogatory and racist today. It is essential to include such articles in our sample, however, so we will take measures to apply the information yielded by the search with sensitivity and respect. We elaborate on this important issue in our Discussion section.

Morbidity was captured by key words such as infection rates, transmission rates, laboratory-confirmed influenza, flu-like illness, and influenza-like illness (ILI). Severe disease was captured by key words such as disease severity, critical illness, critical disease, severe illness, severe disease, hospitalization, patient/hospital admission, and intensive care unit (ICU) admission/treatment. Mortality was captured by key words such as fatal outcome/illness/disease, fatality, lethal outcome/illness/disease, terminal outcome/illness/disease, lethality, and death//mortality rate. All these key words were used in both the pilot and final search as described above. Studies covering both seasonal and pandemic influenza and distinguishing between non-pandemic and pandemic years were included. We also included studies of vaccine efficacy on influenza outcomes that also include Indigenous background as covariate controls. Studies in English, Danish, Norwegian, Swedish, Spanish, and Portuguese were included to generate manageable results, since at least one of the authors was proficient in these languages. Two independent reviewers screen-translated studies independently. In the case of disagreement, the two reviewers discuss the article until consensus is reached.

### Exclusion criteria

Exclusion criteria were as follows:

1. Reviews and theoretical- and policy-oriented papers without original empirical data
2. Studies that included data comparing different Indigenous groups, but no non-Indigenous group(s)
3. Studies on pandemic diseases other than influenza (e.g., COVID-19)
4. Studies on both seasonal and pandemic influenza that did not distinguish between non-pandemic and pandemic years.
5. Studies on influenza vaccine uptake, attitudes towards influenza vaccination, and compliance with (non)pharmaceutical interventions during seasonal influenza or pandemics
6. Case studies or qualitative studies on the associations between Indigenous background and seasonal influenza and pandemic outcomes
7. Studies on social justice and influenza
8. Studies of pandemic influenza preparedness plans

### Data selection and extraction

The following data is extracted from included studies:

1. Article info
  - First author
  - Year published
  - Journal
2. Data sample
  - Country or region of analysis
  - Years up to January 2021 (data search conduction) or pandemic years (1889–1990, 1918–1920, 1957–1958, 1968–1969, 2009–2010)
  - Sample inclusion criteria — i.e., characteristics of Indigenous and non-Indigenous populations (age group/median/average, age, gender, patient group, civilian, military, pregnant, etc.)
  - Sample size
  - Unit of analysis (individuals, households, regions, hospitals, etc.)
  - Data aggregation level (observations of individual units, aggregated units, etc.), e.g., if hospitals are the unit of analysis, does the data used occur at the hospital level, or is it pooled across hospitals?
  - Source of outcome data, e.g., census, routine notification data (e.g., influenza cases reported to a doctor), survey data, and register data
  - If survey or population data had incomplete coverage
    i. Response rate/coverage
    ii. Representativity: Is the sample shown to be representative for the population? I.e., has a non-response analysis been carried out?
3. Outcome variable — Seasonal or pandemic disease outcome ((a) morbidity, (b) hospitalization, (c) mortality)
  - Definition of morbidity: Influenza-like illness (ILI), lab-confirmed infection rates (PCR), transmission rates (reproduction number, R0), and immunity/antibodies towards influenza (HI titer above a certain threshold) due to exposure to the disease and not vaccination
  - Definition of hospitalization: Hospitalized inpatients with PCR, patients admitted to intensive care unit (ICU) or not, mechanically ventilated patients (“lung machines”) or not, inpatients vs outpatients
  - Definition of cause of mortality: Influenza and pneumonia (P&I), excess mortality (P&I, all causes of death, etc.), respiratory diseases, pneumonia, etc.
4. Baseline outcomes (control type), i.e., what was the control group or baseline outcome comparison? (General healthy population, infected patients, the hospitalized, patients with lab-confirmed seasonal influenza)
5. Independent variables of interest — Indigenous group(s) definition versus non-Indigenous groups definition (background/community, area/country, register, self-report, etc.)
6. Statistical methodology
  - Design of study (cross sectional, longitudinal, case-control, cohort studies)
  - Estimation technique (cross tables, correlation analysis, OLS, Poisson regression, logistic regression, Cox regressions, GEE regressions, GLMM models, etc.)
  - Control variables included (e.g., age, gender, SES indices, marital status, preexisting disease, health behavior) in light of sample restrictions (e.g., for pregnant women, gender is not among the controls). SES indices include education, income, crowding, density, deprivation index, unemployment, occupational social class, poverty status, % below poverty level).
  - Reference categories with which all point estimates are compared.
7. Results reported (separate spreadsheet)

### Data synthesis

Before meta-analysis, we plan to conduct narrative analysis based on the main outcomes by Indigenous groups. We plan to use an adapted version [40] of the Effective Public Health Practice Project (EPHPP) checklist for risk of bias and quality assessment. If EPHPP is not precise or sensitive enough for our study results, we will select another appropriate tool for quality assessment. At least two reviewers will assess the overall quality of each study based on study design, sample size, participation rate, attrition, and results. Discussions will be used to resolve discrepancies, if any.

A narrative synthesis will summarize findings according to study characteristics of the included studies, such as seasonal/pandemic years, study region (region/country/hospital), Indigenous and non-Indigenous background, sample size, unit of outcomes, data aggregation level, data sources and type, outcomes, baseline outcomes, design, statistical techniques, controls and whether the study estimates are used in the meta-analysis, and whether (specific) Indigenous background is an independent predictor.

### Meta-analysis

Two meta-analytic techniques will be used to summarize the findings. The studies included assess risk discrepancies in different periods and viral strains among varying Indigenous groups. Considering this, a random effects meta-analytic model will be used to allow for effect heterogeneity. The random effect model allows the study-level effects to differ from each other and estimates the underlying effect variation. The simpler “fixed-effect” model, on the other hand, requires the assumption that the true risk discrepancy estimated (with noise) by the individual studies is the same across all studies. This is unlikely to be appropriate in our context; included studies will use different indicators of Indigenous background and flu outcomes, and their data come from different time periods and countries with different Indigenous groups. The true risk discrepancies are likely to vary across these dimensions. In particular, we expect that the risk discrepancies may be quite different for seasonal and pandemic influenza.

The random effect model will be estimated on the full set of studies with comparable outcome measures (e.g., log odds ratios and relative risk measures). We will also perform split sample analyses to assess how similar results are within subgroups of studies grouped by region, period/pandemic, and Indigenous background.

A risk with such split-sample analyses is that they compare smaller groups, giving less precise estimates and a higher risk of “false-positive” differences in subgroup results. In addition, they do not account for the correlations between the characteristics used to define subgroups. If studies of a specific group tend to come from a specific pandemic period, and if these results also differ from those of other studies, this would affect both the sub-sample analysis comparing different groups, as well as those splitting by period.

Based on prior experience with meta-analysis with a heterogenous sample in a related context [41], we will also use a Bayesian meta-analytic model to assess how estimates vary with study-level indicators and the type of comparisons made. This model allows us to include multiple distinctions simultaneously (e.g., Indigenous group, period, region), using a hierarchical specification to reduce the risk of overfitting. If the evidence indicates that estimates vary no more across study-level indicators than we would expect due to sampling variation, then this will pull the individual indicator coefficients towards zero. The Bayesian model also includes a prior distribution for the parameters, which can be used to express plausible beliefs regarding the parameter values before running the analysis, further reducing the risk that weak tendencies in the data result in inflated estimates. In brief, a hierarchical specification and the use of a prior both function as a form of shrinkage. In other words, the estimated differences between different types of studies are slightly biased towards zero and “no group differences” — but precision is improved since we down-weight implausible and non-credible effect estimates and group differences.

### Colonial bias

Authors of this protocol recognize our non-Indigenous background. We have different national backgrounds (Brazil, Norway, and USA) and recognize that Indigenous participation is important to reduce colonial bias in research. Therefore, we will invite one or two researchers with Indigenous background to participate in the upcoming review and meta-analysis of which this protocol is the basis. They will particularly identify derogatory terms during screening and data extraction, as well as identify colonial bias in interpretations. In line with requirements for all authors, they will be co-authors if they fulfil Vancouver criteria for authorship. If not, they will be thanked in the Acknowledgement section.

## Discussion

To understand the effect of influenza on Indigenous groups, we need to consider more than biology and include embedded social, cultural, economic, and political factors. Both the immediate local community and the distal regional and national environments surrounding Indigenous groups appear to impact the specific pandemic experiences of a particular Indigenous group. All these factors must be taken into account to design culturally appropriate and effective strategies to protect and minimize harm during an influenza pandemic [34]. History can inform us about past experiences with pandemics, which for some groups are connected to painful memories, collective trauma, and even near extinction. Some Amerindian groups have been completely exterminated by outside diseases like smallpox or measles, while others have barely survived after death rates worse than the medieval plague [42]. It is therefore not surprising that new pandemics might revive painful memories, or even trauma, in Indigenous communities.

Some of the past publications on Indigenous groups and influenza, especially those from the earlier pandemics, pose a challenge in their use of terms that are considered as offensive or insensitive today. Inconsistent terms to describe Indigenous groups across time and regions pose a difficulty when it comes to identifying research in Indigenous communities.

Some studies have suggested that co-participation of Indigenous groups in governmental strategies to reduce disease outbreaks may be of special importance, as is trust in regional health services that are culturally sensitive and affordable [43]. A recent review [44] asserted that countries that were successful in protecting their whole population from the COVID-19 pandemic were also those successful in protecting their Indigenous groups. These countries used their knowledge about past pandemics, such as the 2009 influenza pandemic, which disproportionately harmed Indigenous groups, to cooperate with Indigenous representatives in order to engage with Indigenous groups to spread information and reduce infections.

Results from our systematic review and meta-analysis will enable an assessment of the amount of variation in influenza outcomes among Indigenous groups across regions and time since the 1889 pandemic, and this will add to our understanding in the research field. In addition, we expect to identify studies with deviating results (extraordinarily high or low estimations) which may indicate factors implicated in risk disparities for influenza in specific Indigenous groups.

### Supplementary Information

**Additional file 1.** PRISMA-P 2015 Checklist.

**Additional file 2.** Search history.
