# Supplementary material for: Indigenous communities and influenza: protocol for a systematic review and meta-analysis
Source: Syst Rev. 2023 Aug 30;12:151. doi: 10.1186/s13643-023-02319-w (PMC10466723; doi:10.1186/s13643-023-02319-w)
Supplement: Supplementary file 2 — Additional file 2. Search history. [file 13643_2023_2319_MOESM2_ESM.docx]

# **Additional file 2 Search history**

**Database**: Ovid Embase 1974 to 2021 February 05

**Date**: 08.02.2021

**Results**: 1477

| **#** | **Searches** | **Results** |
| --- | --- | --- |
| 1 | influenza/ or influenza a/ or exp "influenza a (h1n1)"/ or exp "influenza a (h2n2)"/ or exp "influenza a (h3n2)"/ or exp "influenza a (h3n8)"/ or influenza b/ or influenza c/ or pandemic influenza/ or seasonal influenza/ or swine influenza/ | 82949 |
| 2 | influenzavirus b/ or influenza b virus/ or influenzavirus c/ or influenza c virus/ or swine influenza virus/ | 2553 |
| 3 | influenza virus/ or influenza a virus/ or influenzavirus a/ or exp "influenza a virus (h1n1)"/ or "influenza a virus (h1n2)"/ or "influenza a virus (h2n2)"/ or "influenza a virus (h3n2)"/ or "influenza a virus (h3n8)"/ | 28633 |
| 4 | Hong Kong influenza/ or Asian influenza/ or Spanish influenza/ or 1889 Russian influenza/ or 2009 H1N1 influenza/ or pandemic influenza/ or seasonal influenza/ or swine influenza/ or 1977 russian influenza/ | 14108 |
| 5 | Influenza virus A/ | 19572 |
| 6 | Influenza virus B/ | 5423 |
| 7 | Influenza virus C/ | 556 |
| 8 | Orthomyxoviridae/ | 434 |
| 9 | orthomyxovirus infection/ | 1891 |
| 10 | (influenza or influenzas or influenzavirus or flu or flus or H1N1 or H1N2 or H2N2 or H3N2 or H3N8).tw,kw. | 129836 |
| 11 | (PH1N1 or H1N1pdm09 or H1N1p or A?H1N1*).tw,kw. | 1545 |
| 12 | ((Russian or Spanish or Asian or Hong Kong or Mexican) adj4 (pandemic* or epidemic*)).tw,kw. | 911 |
| 13 | (("1889" or 1889-90 or 1889-1890 or 1889-91 or 1889-1891 or 1889-92 or 1889-1892 or 1889-93 or 1889-1893 or 1889-94 or 1889-1894 or 1918-19 or 1918-1919 or 1918-20 or 1918-1920 or "1957" or "1958" or 1957-58 or 1957-1958 or "1968" or 1968-69 or 1968-1969 or 1968-70 or 1968-1970 or "1969" or 1969-70 or 1969-1970 or "1970" or "2009" or 2009-10 or 2009-2010) adj7 (pandemic* or epidemic* or outbreak*)).tw,kw. | 10619 |
| 14 | or/1-13 | 155871 |
| 15 | indigenous people/ or Alaska Native/ or American Indian/ or Canadian Aboriginal/ or First Nation/ or Indigenous Australian/ or ancestry group/ or Eskimo/ or Inuit/ or "Yupik (people)"/ or indigenous health care/ or taiwanese aborigine/ or na-dene people/ or "athabaskan (people)"/ or "haida (people)"/ or "tlingit (people)"/ or uralic people/ or samoyedic people/ or "nenets (people)"/ or "nganasan (people)"/ or finno-ugric people/ or "karelian (people)"/ or "khanty (people)"/ or "komi (people)"/ or "mansi (people)"/ or "sami (people)"/ or "udmurt (people)"/ or tai-kadai people/ or tai people/ or "zhuang (people)"/ or sino-tibetan people/ or tibeto-burman people/ or sino-tibetan people/ or "bamar (people)"/ or "jingpo (people)"/ or "karen (people)"/ or "meitei (people)"/ or "naga (people)"/ or "newar (people)"/ or "tamang (people)"/ or exp pygmy/ or papuan people/ or "asmat (people)"/ or paleosiberian people/ or chukotko-kamchatkan people/ or "chukchi (people)"/ or "koryak (people)"/ or nilo-saharan people/ or "fur (people)"/ or "kanuri (people)"/ or "nubian (people)"/ or nilote/ or "dinka (people)"/ or "kalenjin (people)"/ or exp "luo (people)"/ or "maasai (people)"/ or "nuer (people)"/ or "samburu (people)"/ or niger-congo people/ or "akan (people)"/ or exp bantu people/ or "dogon (people)"/ or exp gur people/ or "igbo (people)"/ or "ijaw (people)"/ or exp mande people/ or "nuba (people)"/ or exp senegambian people/ or "yoruba (people)"/ or negrito/ or "aeta (people)"/ or "andamanese (people)"/ or "mamanwa (people)"/ or khoisan/ or khoikhoi/ or san people/ or hmong-mien people/ or "hmong (people)"/ or "mien (people)"/ or eskimo-aleut people/ or "inupiat (people)"/ or "hadza (people)"/ or dravidian people/ or kannadiga/ or "malayalam (people)"/ or "tamil (people)"/ or "telugu (people)"/ or austronesian people/ or "atayal (people)"/ or "bunun (people)"/ or "paiwan (people)"/ or "yami (people)"/ or malayo-polynesian people/ or "batak (people)"/ or "chamorro (people)"/ or "malay (people)"/ or "maori (people)"/ or native hawaiian/ or "samoan (people)"/ or austroasiatic people/ or mon-khmer people/ or "khmer (people)"/ or "kinh (people)"/ or "nicobarese (people)"/ or orang asli/ or "munda (people)"/ or australian aborigine/ or "tiwi (people)"/ or north-central amerind people/ or "sioux (people)"/ or "lakota (people)"/ or uto-aztecan people/ or north-central amerind people/ or "hopi (people)"/ or "huichol (people)"/ or "mayo (people)"/ or exp nahua/ or "pima (people)"/ or "tarahumara (people)"/ or "tepehuano (people)"/ or tohono o'odham/ or "yaqui (people)"/ or penutian people/ or "zuni (people)"/ or "muscogee (people)"/ or "choctaw (people)"/ or "seminole (people)"/ or oto-manguean people/ or "mazahua (people)"/ or "mazatec (people)"/ or "mixtec (people)"/ or "otomi (people)"/ or "zapotec (people)"/ or "iroquois (people)"/ or "cherokee (people)"/ or "mohawk (people)"/ or caddoan people/ or "arikara (people)"/ or "maya (people)"/ or "ojibway (people)"/ or algonkian people/ or "algonquin (people)"/ or "chippewa (people)"/ or "oji-cree (people)"/ or "cree (people)"/ or southern amerind people/ or andean amerind people/ or "aymara (people)"/ or "mapuche (people)"/ or "quechua (people)"/ or "waorani (people)"/ or arawak/ or "carib (people)"/ or chibchan-paezan people/ or "chibcha (people)"/ or "purepecha (people)"/ or "warao (people)"/ or "yanomami (people)"/ or "jivaro (people)"/ or "kaingang (people)"/ or "piaroa (people)"/ or "ticuna (people)"/ or tupi-guarani people/ or "guarani (people)"/ or "tupi (people)"/ or mongolic people/ | 42050 |
| 16 | altaic people/ or "buryat (people)"/ or "daur (people)"/ or "kalmyk (people)"/ or "mongolian (people)"/ or tungusic people/ or "even (people)"/ or "evenk (people)"/ or "manchu (people)"/ or "oroqen (people)"/ or turkic people/ or "azeri (people)"/ or "bashkir (people)"/ or "kazakh (people)"/ or "kyrgyz (people)"/ or "tatar (people)"/ or "uygur (people)"/ or "yakut (people)"/ or afro-asiatic people/ or berber/ or tuareg/ or chadic people/ or "hausa (people)"/ or cushitic people/ or "afar (people)"/ or "oromo (people)"/ or "somali (people)"/ or semitic people/ or "amhara (people)"/ or druze/ or bedouin/ | 2269 |
| 17 | (Indigen* or tribal or tribe* or first nation* or native* or nativity or american indian* or aborigin*).tw,kw. | 332365 |
| 18 | (eskimo$2 or inuit$2 or innu$2 or inupiaq$2 or alutiiq$2 or unangax$2 or yup?ik$2 or cup?ik$2 or athabascan$2 or labrador$2 or newfoundland$2 or na-dene$2 or athabaskan$2 or haida$2 or tlingit$2 or eyak$2 or tsimshian$2 or uralic$2 or samoyed$2 or nenets$2 or nganasan$2 or finno-ugric$2 or karelian$2 or khanty$2 or komi$2 or mansi$2 or sami$2 or udmurt$2 or tai-kadai$2 or tai or tais or zhuang$2 or sino-tibetan$2 or tibeto-burman$2 or bamar$2 or jingpo$2 or karen$2 or meitei$2 or naga$2 or newar$2 or tamang$2 or pygmy$2 or pygmies or papuan$2 or asmat$2 or paleosiberian$2 or chukotko-kamchatkan$2 or chukchi$2 or koryak$2 or nilo-saharan$2 or kanuri$2 or nubian$2 or nilote$2 or dinka$2 or kalenjin$2 or luo$2 or maasai$2 or nuer$2 or samburu$2 or niger-congo$2 or akan$2 or bantu$2 or dogon$2 or gur or gurs or igbo$2 or ijaw$2 or mande$2 or nuba$2 or senegambian$2 or yoruba$2 or negrito$2 or aeta$2 or andamanese$2 or mamanwa$2 or khoisan$2 or khoikhoi$2 or san people$2 or hmong$2 or mien$2 or aleut$2 or inupiat$2 or hadza$2 or dravidian$2 or kannadiga$2 or malayalam$2 or tamil$2 or telugu$2 or austronesian$2 or atayal$2 or bunun$2 or paiwan$2 or yami$2 or malayo-polynesian$2 or batak$2 or chamorro$2 or malay$2 or maori$2 or samoan$2 or austroasiatic$2 or khmer$2 or kinh$2 or nicobarese$2 or orang asli$2 or munda$2 or torres strait$2 or pacific islander$2 or pacific people$2 or tiwi$2 or amerind$2 or sioux$2 or lakota$2 or uto-aztecan$2 or huichol$2 or nahua$2 or pima$2 or tarahumara$2 or tepehuano$2 or tohono o?odham$2 or yaqui$2 or penutian$2 or zuni$2 or muscogee$2 or choctaw$2 or seminole$2 or oto-manguean$2 or mazahua$2 or mazatec$2 or mixtec$2 or otomi$2 or zapotec$2 or iroquois$2 or cherokee$2 or mohawk$2 or caddoan$2 or arikara$2 or maya$2 or ojibway$2 or algonkian$2 or algonquin$2 or chippewa$2 or cree$2 or aymara$2 or mapuche$2 or quechua$2 or waorani$2 or arawak$2 or chibchan-paezan$2 or chibcha$2 or purepecha or warao$2 or yanomami$2 or jivaro$2 or kaingang$2 or piaroa$2 or ticuna$2 or guarani$2 or tupi$2 or mongolic$2 or altaic$2 or buryat$2 or daur$2 or kalmyk$2 or mongolian$2 or tungusic$2 or evenk$2 or manchu$2 or oroqen$2 or turkic$2 or azeri$2 or bashkir$2 or kazakh$2 or kyrgyz$2 or tatar$2 or uygur$2 or yakut$2 or afro-asiatic$2 or berber$2 or tuareg$2 or chadic$2 or hausa$2 or cushitic$2 or oromo$2 or somali$2 or amhara$2 or druze$2 or bedouin$2 or roma$2 or gypsy or gypsies).tw,kw. | 117254 |
| 19 | or/15-18 | 450675 |
| 20 | 14 and 19 | 2078 |
| 21 | (14 and 19) not ((exp animal/ or exp invertebrate/ or nonhuman/ or animal experiment/ or animal tissue/ or animal model/ or exp plant/ or exp fungus/) not (exp human/ or human tissue/)) | 1477 |

**Database**: Ovid MEDLINE(R) ALL <1946 to February 05, 2021>

**Date**: 08.02.2021

**Results**: 1753

| **#** | **Searches** | **Results** |
| --- | --- | --- |
| 1 | orthomyxoviridae/ or influenzavirus a/ or influenza a virus/ or influenza a virus, h1n1 subtype/ or influenza a virus, h1n2 subtype/ or influenza a virus, h2n2 subtype/ or influenza a virus, h3n2 subtype/ or influenza a virus, h3n8 subtype/ or influenzavirus b/ or influenza b virus/ or influenzavirus c/ | 51114 |
| 2 | Influenza Pandemic, 1918-1919/ | 161 |
| 3 | Influenza, Human/ | 50657 |
| 4 | (influenza or influenzas or influenzavirus or flu or flus or H1N1 or H1N2 or H2N2 or H3N2 or H3N8).tw,kw,kf. | 111999 |
| 5 | (PH1N1 or H1N1pdm09 or H1N1p or A?H1N1*).tw,kw,kf. | 1314 |
| 6 | ((Russian or Spanish or Asian or Hong Kong or Mexican) adj4 (pandemic* or epidemic*)).tw,kw,kf. | 911 |
| 7 | (("1889" or 1889-90 or 1889-1890 or 1889-91 or 1889-1891 or 1889-92 or 1889-1892 or 1889-93 or 1889-1893 or 1889-94 or 1889-1894 or 1918-19 or 1918-1919 or 1918-20 or 1918-1920 or "1957" or "1958" or 1957-58 or 1957-1958 or "1968" or 1968-69 or 1968-1969 or 1968-70 or 1968-1970 or "1969" or 1969-70 or 1969-1970 or "1970" or "2009" or 2009-10 or 2009-2010) adj7 (pandemic* or epidemic* or outbreak*)).tw,kw,kf. | 8730 |
| 8 | or/1-7 | 122009 |
| 9 | african continental ancestry group/ | 38198 |
| 10 | american native continental ancestry group/ | 473 |
| 11 | alaska natives/ | 397 |
| 12 | indians, central american/ | 543 |
| 13 | indians, north american/ | 14616 |
| 14 | indians, south american/ | 3641 |
| 15 | inuits/ | 3938 |
| 16 | asian continental ancestry group/ | 67253 |
| 17 | european continental ancestry group/ | 67670 |
| 18 | oceanic ancestry group/ | 10595 |
| 19 | indigenous peoples/ | 376 |
| 20 | roma/ | 940 |
| 21 | (Indigen* or tribal or tribe* or first nation* or native* or nativity or american indian* or aborigin*).tw,kw,kf. | 287652 |
| 22 | (eskimo$2 or inuit$2 or innu$2 or inupiaq$2 or alutiiq$2 or unangax$2 or yup?ik$2 or cup?ik$2 or athabascan$2 or labrador$2 or newfoundland$2 or na-dene$2 or athabaskan$2 or haida$2 or tlingit$2 or eyak$2 or tsimshian$2 or uralic$2 or samoyed$2 or nenets$2 or nganasan$2 or finno-ugric$2 or karelian$2 or khanty$2 or komi$2 or mansi$2 or sami$2 or udmurt$2 or tai-kadai$2 or tai or tais or zhuang$2 or sino-tibetan$2 or tibeto-burman$2 or bamar$2 or jingpo$2 or karen$2 or meitei$2 or naga$2 or newar$2 or tamang$2 or pygmy$2 or pygmies or papuan$2 or asmat$2 or paleosiberian$2 or chukotko-kamchatkan$2 or chukchi$2 or koryak$2 or nilo-saharan$2 or kanuri$2 or nubian$2 or nilote$2 or dinka$2 or kalenjin$2 or luo$2 or maasai$2 or nuer$2 or samburu$2 or niger-congo$2 or akan$2 or bantu$2 or dogon$2 or gur or gurs or igbo$2 or ijaw$2 or mande$2 or nuba$2 or senegambian$2 or yoruba$2 or negrito$2 or aeta$2 or andamanese$2 or mamanwa$2 or khoisan$2 or khoikhoi$2 or san people$2 or hmong$2 or mien$2 or aleut$2 or inupiat$2 or hadza$2 or dravidian$2 or kannadiga$2 or malayalam$2 or tamil$2 or telugu$2 or austronesian$2 or atayal$2 or bunun$2 or paiwan$2 or yami$2 or malayo-polynesian$2 or batak$2 or chamorro$2 or malay$2 or maori$2 or samoan$2 or austroasiatic$2 or khmer$2 or kinh$2 or nicobarese$2 or orang asli$2 or munda$2 or torres strait$2 or pacific islander$2 or pacific people$2 or tiwi$2 or amerind$2 or sioux$2 or lakota$2 or uto-aztecan$2 or huichol$2 or nahua$2 or pima$2 or tarahumara$2 or tepehuano$2 or tohono o?odham$2 or yaqui$2 or penutian$2 or zuni$2 or muscogee$2 or choctaw$2 or seminole$2 or oto-manguean$2 or mazahua$2 or mazatec$2 or mixtec$2 or otomi$2 or zapotec$2 or iroquois$2 or cherokee$2 or mohawk$2 or caddoan$2 or arikara$2 or maya$2 or ojibway$2 or algonkian$2 or algonquin$2 or chippewa$2 or cree$2 or aymara$2 or mapuche$2 or quechua$2 or waorani$2 or arawak$2 or chibchan-paezan$2 or chibcha$2 or purepecha or warao$2 or yanomami$2 or jivaro$2 or kaingang$2 or piaroa$2 or ticuna$2 or guarani$2 or tupi$2 or mongolic$2 or altaic$2 or buryat$2 or daur$2 or kalmyk$2 or mongolian$2 or tungusic$2 or evenk$2 or manchu$2 or oroqen$2 or turkic$2 or azeri$2 or bashkir$2 or kazakh$2 or kyrgyz$2 or tatar$2 or uygur$2 or yakut$2 or afro-asiatic$2 or berber$2 or tuareg$2 or chadic$2 or hausa$2 or cushitic$2 or oromo$2 or somali$2 or amhara$2 or druze$2 or bedouin$2 or roma$2 or gypsy or gypsies).tw,kw,kf. | 95513 |
| 23 | or/9-22 | 516401 |
| 24 | 8 and 23 | 2087 |
| 25 | (8 and 23) not ((exp animal/ or exp invertebrate/ or animal experiment/ or animal model/ or exp plant/ or exp fungus/) not exp human/) | 1753 |

**Database**: Web of Science

**Date**: 08.02.2021

**Results**: 1654

|  | Query | Results |
| --- | --- | --- |
| 1 | TS=((Influenza or influenzas or influenzavirs or flu or flus or H1N1 or H1N2 or H2N2 or H3N2 or H3N8)  Indexes=SCI-EXPANDED, SSCI, ESCI Timespan=1987-2021 | [114,365](https://apps.webofknowledge.com/summary.do?product=WOS&doc=1&qid=23&SID=F6kEtmARhTVp6LMP2Xv&search_mode=AdvancedSearch&update_back2search_link_param=yes) |
| 2 | TS=(PH1N1 or H1N1pdm09 or H1N1p)  Indexes=SCI-EXPANDED, SSCI, ESCI Timespan=1987-2021 | [1,033](https://apps.webofknowledge.com/summary.do?product=WOS&doc=1&qid=3&SID=F6kEtmARhTVp6LMP2Xv&search_mode=AdvancedSearch&update_back2search_link_param=yes) |
| 3 | TS=((Russian or Spanish or Asian or "Hong Kong" or Mexican) NEAR/3 (pandemic* or epidemic*) )  Indexes=SCI-EXPANDED, SSCI, ESCI Timespan=1987-2021 | [866](https://apps.webofknowledge.com/summary.do?product=WOS&doc=1&qid=27&SID=F6kEtmARhTVp6LMP2Xv&search_mode=AdvancedSearch&update_back2search_link_param=yes) |
| 4 | TS=(("1889" or 1889-90 or 1889-1890 or 1889-91 or 1889-1891 or 1889-92 or 1889-1892 or 1889-93 or 1889-1893 or 1889-94 or 1889-1894 or 1918-19 or 1918-1919 or 1918-20 or 1918-1920 or "1957" or "1958" or 1957-58 or 1957-1958 or "1968" or 1968-69 or 1968-1969 or 1968-70 or 1968-1970 or "1969" or 1969-70 or 1969-1970 or "1970" or "2009" or 2009-10 or 2009-2010) NEAR/6 (pandemic* or epidemic* or outbreak*) )  Indexes=SCI-EXPANDED, SSCI, ESCI Timespan=1987-2021 | [8,357](https://apps.webofknowledge.com/summary.do?product=WOS&doc=1&qid=30&SID=F6kEtmARhTVp6LMP2Xv&search_mode=AdvancedSearch&update_back2search_link_param=yes) |
| 5 | #4 OR #3 OR #2 OR #1  Indexes=SCI-EXPANDED, SSCI, ESCI Timespan=1987-2021 | [116,216](https://apps.webofknowledge.com/summary.do?product=WOS&doc=1&qid=31&SID=F6kEtmARhTVp6LMP2Xv&search_mode=CombineSearches&update_back2search_link_param=yes) |
| 6 | TS=(Indigen* or tribal or tribe* or "first nation*" or native* or nativity or "american indian*" or aborigin*)  Indexes=SCI-EXPANDED, SSCI, ESCI Timespan=1987-2021 | \|  \| [455,839](https://apps.webofknowledge.com/summary.do?product=WOS&doc=1&qid=9&SID=F6kEtmARhTVp6LMP2Xv&search_mode=AdvancedSearch&update_back2search_link_param=yes) \| \| --- \| --- \| |
| 7 | TS=((eskimo or eskimos or inuit or inuits or innu or innus or Inupiaq or inuqiaqs or alutiiq or alutiiqs or unangax or unangaxs or yup?ik or yup?iks or cup?ik or cup?iks or athabascan or athabascans or Labrador or Newfoundlander or Newfoundlanders or Newfoundland or “na-dene” or “na-denes” or athabaskan or athabaskans or haida or haidas or tlingit or tlingits or eyak or eyaks or tsimshian or tsimshians or uralic or uralics or samoyed or samoyeds or nenets or nganasan or nganasans or “finno-ugric” or karelian or karelians or khanty or khanties or komi or komis or mansi or mansis or sami or udmurt or udmurts or “tai-kadai” or “tai-kadais” or tai or tais or zhuang or “sino-tibetan” or “sino-tibetans” or “tibeto-burman” or “tibeto-burmans” or bamar or bamars or jingpo or jingpos or karen or karens or meitei or meiteis or naga or newar or tamang or pygmy* or pygmies or papuan or papuans or asmar or paleosiberian or paleosiberians or “chukotko-kamchatkan” or “chukotko-kamchatkans” or chukchi or koryak or “nilo-saharan” or “nilo-saharans” or gur or gurs or igbo or igbos or ijaw or jiaws or mande or mandes or nuba or nubas or senegambian or senegambians or yoruba or yorubas or negrito or negritos or aeta or aetas or andamanese or mamanwa or mamanwas or khoisan or khoisans or khoikhoi or khoikhois or “san people” or “san peoples” or hmong or hmongs or mien or miens or aleut or aleuts or inupiat or inupitats or hadza or hadzas or dravidian or dravidians or kannadiga or kannadigas or malayalam or malayalams or tamil or tamils or telugu or telugus or austronesian or austronesians or atayal or atayals or bunun or bununs or paiwan or paiwans or yami or yamis or “malayo-polynesian” or “malayo-polynesians” or batak or bataks or chamorro or chamorros or malay or malays or maori or maoris or samoan or samoans or austroasiatic or khmer or khmers or kinh or kinhs or nicobarese or “orang asli” or munda or mundas or “torres strait” or “pacific islanders” or “pacific islander” or “pacific people” or “pacific peoples” or tiwi or tiwis or amerind or amerinds or sioux or siouxs or lakota or lakotas or “uto-aztecan” or “uto-aztecans” or huichol or huichols or nahua or nahuas or pima or pimas or tarahumara or tarahumaras or tepehuano or tepehuanos or “tohono o?odham” or “tohono o?odhams” or yaqui or yaquis or penutian or penutians or zuni or zunis or muscogee or muscogees or choctaw or choctaws or seminole or seminoles or “oto-manguean” or “oto-mandueans” or mazahua or mazahuas or mazatec or mazatecs or mixtec or mixtexs or otomi or otomis or zapotec or zapotecs or iroquois or cherokee or cherokees or mohawk or mohawks or caddoan or caddoans or arikara or arikaras or maya or mayas or ojibway orojibways or algonkian or algonkians or algonquin or algonquins or chippewa or chippewas or cree or crees or aymara or aymaras or mapuche or mapuches or quechua or quechuas or waorani or waoranis or arawak or arawaks or “chibchan-paezan” or “chibchan-paezans” or chibcha or chibchas or purepecha or purepechas or warao or waraos or yanomami or yanomamis or jivaro or jivaros or kaingang or kainangs or piaroa or piaroas or ticuna or ticunas or guarani or guaranis or tupi or tupis or mongolic or altaic or buryat or buryats or daur or daurs or kalmyk or kalmyks or mongolian or mongolians or tungusic or tungusics or evenk or evenks or manchu or manchus or oroqen or oroqens or turkic or turkics or azeri or azeris or bashkir or bashkirs or kazakh or kazakhs or kyrgyz or tatar or tatars or uygur or uygurs or yakut or yakuts or “afro-asiatic*” or berber or berbers or tuareg or tuaregs or chadic or chadics or hausa or hausas or cushitic or oromo or oromos or somali or somalis or amhara or amharas or druze or druzes or bedouin or bedouins or gypsy or gypsies or roma or romas) | [126,880](https://apps.webofknowledge.com/summary.do?product=WOS&doc=1&qid=17&SID=F6kEtmARhTVp6LMP2Xv&search_mode=AdvancedSearch&update_back2search_link_param=yes) |
| 8 | #7 OR #6  Indexes=SCI-EXPANDED, SSCI, ESCI Timespan=1987-2021 | [565,543](https://apps.webofknowledge.com/summary.do?product=WOS&doc=1&qid=18&SID=F6kEtmARhTVp6LMP2Xv&search_mode=CombineSearches&update_back2search_link_param=yes) |
| 9 | #8 AND #5  Indexes=SCI-EXPANDED, SSCI, ESCI Timespan=1987-2021 | 1654 |

**Database**: Cinahl via EBSCOhost

**Date**: 08.02.2021

**Results**: 298

| # | Query | Results |
| --- | --- | --- |
| S1 | (MH "Influenza+") OR (MH "Influenza A Virus+") OR (MH "Influenza, Pandemic (H1N1) 2009") OR (MH "Influenza, Avian") OR (MH "Influenzavirus C") OR (MH "Influenza B Virus") OR (MH "Influenza A Virus, H1N1 Subtype") OR (MH "Influenza, Swine") OR (MH "Influenza, Seasonal") OR (MH "Influenza, Human+") OR (MH "Influenza A Virus, H3N2 Subtype") OR (MH "Orthomyxoviridae+") OR (MH "Influenzavirus A+") | 21,767 |
| S2 | TI(influenza or influenzas or influenzavirus or flu or flus or H1N1 or H1N2 or H2N2 or H3N2 or H3N8 or PH1N1 or H1N1pdm09 or H1N1p) OR AB (influenza or influenzas or influenzavirus or flu or flus or H1N1 or H1N2 or H2N2 or H3N2 or H3N8 or PH1N1 or H1N1pdm09 or H1N1p) | 26,558 |
| S3 | TI ((Russian or Spanish or Asian or "Hong Kong" or Mexican) N3 (pandemic* or epidemic*)) OR AB ((Russian or Spanish or Asian or "Hong Kong" or Mexican) N3 (pandemic* or epidemic*)) | 187 |
| S4 | TI ((("1889" or 1889-90 or 1889-1890 or 1889-91 or 1889-1891 or 1889-92 or 1889-1892 or 1889-93 or 1889-1893 or 1889-94 or 1889-1894 or 1918-19 or 1918-1919 or 1918-20 or 1918-1920 or "1957" or "1958" or 1957-58 or 1957-1958 or "1968" or 1968-69 or 1968-1969 or 1968-70 or 1968-1970 or "1969" or 1969-70 or 1969-1970 or "1970" or "2009" or 2009-10 or 2009-2010) N6 (pandemic* or epidemic* or outbreak*))) OR AB ((("1889" or 1889-90 or 1889-1890 or 1889-91 or 1889-1891 or 1889-92 or 1889-1892 or 1889-93 or 1889-1893 or 1889-94 or 1889-1894 or 1918-19 or 1918-1919 or 1918-20 or 1918-1920 or "1957" or "1958" or 1957-58 or 1957-1958 or "1968" or 1968-69 or 1968-1969 or 1968-70 or 1968-1970 or "1969" or 1969-70 or 1969-1970 or "1970" or "2009" or 2009-10 or 2009-2010) N6 (pandemic* or epidemic* or outbreak*))) | 2,060 |
| S5 | S1 OR S2 OR S3 OR S4 | 31,505 |
| S6 | (MH "Indigenous Health") OR (MH "Indigenous Peoples+") OR (MH "Aboriginal Canadians") OR (MH "First Nations of Canada") OR (MH "Eskimos") OR (MH "Inuit") OR (MH "First Nations of Australia") OR (MH "Aboriginal Australians") OR (MH "Torres Strait Islanders") OR (MH "Maori") OR (MH "Native Americans") OR (MH "Gypsies") | 20,985 |
| S7 | TI (Indigen* or tribal or tribe* or "first nation*" or native* or nativity or "american indian*" or aborigin*) OR AB (Indigen* or tribal or tribe* or "first nation*" or native* or nativity or "american indian*" or aborigin*) | 37,456 |
| S8 | TI (eskimo or eskimos or inuit or inuits or innu or innus or Inupiaq or inuqiaqs or alutiiq or alutiiqs or unangax or unangaxs or yup?ik or yup?iks or cup?ik or cup?iks or athabascan or athabascans or Labrador or Newfoundlander or Newfoundlanders or Newfoundland or “na-dene” or “na-denes” or athabaskan or athabaskans or haida or haidas or tlingit or tlingits or eyak or eyaks or tsimshian or tsimshians or uralic or uralics or samoyed or samoyeds or nenets or nganasan or nganasans or “finno-ugric” or karelian or karelians or khanty or khanties or komi or komis or mansi or mansis or sami or udmurt or udmurts or “tai-kadai” or “tai-kadais” or tai or tais or zhuang or “sino-tibetan” or “sino-tibetans” or “tibeto-burman” or “tibeto-burmans” or bamar or bamars or jingpo or jingpos or karen or karens or meitei or meiteis or naga or newar or tamang or pygmy* or pygmies or papuan or papuans or asmar or paleosiberian or paleosiberians or “chukotko-kamchatkan” or “chukotko-kamchatkans” or chukchi or koryak or “nilo-saharan” or “nilo-saharans” or gur or gurs or igbo or igbos or ijaw or jiaws or mande or mandes or nuba or nubas or senegambian or senegambians or yoruba or yorubas or negrito or negritos or aeta or aetas or andamanese or mamanwa or mamanwas or khoisan or khoisans or khoikhoi or khoikhois or “san people” or “san peoples” or hmong or hmongs or mien or miens or aleut or aleuts or inupiat or inupitats or hadza or hadzas or dravidian or dravidians or kannadiga or kannadigas or malayalam or malayalams or tamil or tamils or telugu or telugus or austronesian or austronesians or atayal or atayals or bunun or bununs or paiwan or paiwans or yami or yamis or “malayo-polynesian” or “malayo-polynesians” or batak or bataks or chamorro or chamorros or malay or malays or maori or maoris or samoan or samoans or austroasiatic or khmer or khmers or kinh or kinhs or nicobarese or “orang asli” or munda or mundas or “torres strait” or “pacific islanders” or “pacific islander” or “pacific people” or “pacific peoples” or tiwi or tiwis or amerind or amerinds or sioux or siouxs or lakota or lakotas or “uto-aztecan” or “uto-aztecans” or huichol or huichols or nahua or nahuas or pima or pimas or tarahumara or tarahumaras or tepehuano or tepehuanos or “tohono o?odham” or “tohono o?odhams” or yaqui or yaquis or penutian or penutians or zuni or zunis or muscogee or muscogees or choctaw or choctaws or seminole or seminoles or “oto-manguean” or “oto-mandueans” or mazahua or mazahuas or mazatec or mazatecs or mixtec or mixtexs or otomi or otomis or zapotec or zapotecs or iroquois or cherokee or cherokees or mohawk or mohawks or caddoan or caddoans or arikara or arikaras or maya or mayas or ojibway orojibways or algonkian or algonkians or algonquin or algonquins or chippewa or chippewas or cree or crees or aymara or aymaras or mapuche or mapuches or quechua or quechuas or waorani or waoranis or arawak or arawaks or “chibchan-paezan” or “chibchan-paezans” or chibcha or chibchas or purepecha or purepechas or warao or waraos or yanomami or yanomamis or jivaro or jivaros or kaingang or kainangs or piaroa or piaroas or ticuna or ticunas or guarani or guaranis or tupi or tupis or mongolic or altaic or buryat or buryats or daur or daurs or kalmyk or kalmyks or mongolian or mongolians or tungusic or tungusics or evenk or evenks or manchu or manchus or oroqen or oroqens or turkic or turkics or azeri or azeris or bashkir or bashkirs or kazakh or kazakhs or kyrgyz or tatar or tatars or uygur or uygurs or yakut or yakuts or “afro-asiatic*” or berber or berbers or tuareg or tuaregs or chadic or chadics or hausa or hausas or cushitic or oromo or oromos or somali or somalis or amhara or amharas or druze or druzes or bedouin or bedouins or gypsy or gypsies or roma or romas) OR AB (eskimo or eskimos or inuit or inuits or innu or innus or Inupiaq or inuqiaqs or alutiiq or alutiiqs or unangax or unangaxs or yup?ik or yup?iks or cup?ik or cup?iks or athabascan or athabascans or Labrador or Newfoundlander or Newfoundlanders or Newfoundland or “na-dene” or “na-denes” or athabaskan or athabaskans or haida or haidas or tlingit or tlingits or eyak or eyaks or tsimshian or tsimshians or uralic or uralics or samoyed or samoyeds or nenets or nganasan or nganasans or “finno-ugric” or karelian or karelians or khanty or khanties or komi or komis or mansi or mansis or sami or udmurt or udmurts or “tai-kadai” or “tai-kadais” or tai or tais or zhuang or “sino-tibetan” or “sino-tibetans” or “tibeto-burman” or “tibeto-burmans” or bamar or bamars or jingpo or jingpos or karen or karens or meitei or meiteis or naga or newar or tamang or pygmy* or pygmies or papuan or papuans or asmar or paleosiberian or paleosiberians or “chukotko-kamchatkan” or “chukotko-kamchatkans” or chukchi or koryak or “nilo-saharan” or “nilo-saharans” or gur or gurs or igbo or igbos or ijaw or jiaws or mande or mandes or nuba or nubas or senegambian or senegambians or yoruba or yorubas or negrito or negritos or aeta or aetas or andamanese or mamanwa or mamanwas or khoisan or khoisans or khoikhoi or khoikhois or “san people” or “san peoples” or hmong or hmongs or mien or miens or aleut or aleuts or inupiat or inupitats or hadza or hadzas or dravidian or dravidians or kannadiga or kannadigas or malayalam or malayalams or tamil or tamils or telugu or telugus or austronesian or austronesians or atayal or atayals or bunun or bununs or paiwan or paiwans or yami or yamis or “malayo-polynesian” or “malayo-polynesians” or batak or bataks or chamorro or chamorros or malay or malays or maori or maoris or samoan or samoans or austroasiatic or khmer or khmers or kinh or kinhs or nicobarese or “orang asli” or munda or mundas or “torres strait” or “pacific islanders” or “pacific islander” or “pacific people” or “pacific peoples” or tiwi or tiwis or amerind or amerinds or sioux or siouxs or lakota or lakotas or “uto-aztecan” or “uto-aztecans” or huichol or huichols or nahua or nahuas or pima or pimas or tarahumara or tarahumaras or tepehuano or tepehuanos or “tohono o?odham” or “tohono o?odhams” or yaqui or yaquis or penutian or penutians or zuni or zunis or muscogee or muscogees or choctaw or choctaws or seminole or seminoles or “oto-manguean” or “oto-mandueans” or mazahua or mazahuas or mazatec or mazatecs or mixtec or mixtexs or otomi or otomis or zapotec or zapotecs or iroquois or cherokee or cherokees or mohawk or mohawks or caddoan or caddoans or arikara or arikaras or maya or mayas or ojibway orojibways or algonkian or algonkians or algonquin or algonquins or chippewa or chippewas or cree or crees or aymara or aymaras or mapuche or mapuches or quechua or quechuas or waorani or waoranis or arawak or arawaks or “chibchan-paezan” or “chibchan-paezans” or chibcha or chibchas or purepecha or purepechas or warao or waraos or yanomami or yanomamis or jivaro or jivaros or kaingang or kainangs or piaroa or piaroas or ticuna or ticunas or guarani or guaranis or tupi or tupis or mongolic or altaic or buryat or buryats or daur or daurs or kalmyk or kalmyks or mongolian or mongolians or tungusic or tungusics or evenk or evenks or manchu or manchus or oroqen or oroqens or turkic or turkics or azeri or azeris or bashkir or bashkirs or kazakh or kazakhs or kyrgyz or tatar or tatars or uygur or uygurs or yakut or yakuts or “afro-asiatic*” or berber or berbers or tuareg or tuaregs or chadic or chadics or hausa or hausas or cushitic or oromo or oromos or somali or somalis or amhara or amharas or druze or druzes or bedouin or bedouins or gypsy or gypsies or roma or romas) | 20,367 |
| S9 | S6 OR S7 OR S8 | 61,081 |
| S10 | S5 AND S9 | 298 |

**Database**: SocINDEX via EBSCOhost

**Date**: 08.02.2021

**Results**: 80

| # | Query | Results |
| --- | --- | --- |
| S1 | DE "INFLUENZA" | 404 |
| S2 | TI(influenza or influenzas or flu or flus or H1N1 or H1N2 or H2N2 or H3N2 or H3N8) OR AB ((influenza or influenzas or flu or flus or H1N1 or H1N2 or H2N2 or H3N2 or H3N8) OR SU (influenza or influenzas or flu or flus or H1N1 or H1N2 or H2N2 or H3N2 or H3N8) OR KW (influenza or influenzas or flu or flus or H1N1 or H1N2 or H2N2 or H3N2 or H3N8) | 1,937 |
| S3 | TI ( (PH1N1 or H1N1pdm09 or H1N1p) ) OR AB ( (PH1N1 or H1N1pdm09 or H1N1p) ) OR SU (PH1N1 or H1N1pdm09 or H1N1p) ) OR KW ( (PH1N1 or H1N1pdm09 or H1N1p) ) | 10 |
| S4 | TI ( ((Russian or Spanish or Asian or "Hong Kong" or Mexican) N3 (pandemic* or epidemic*)) ) OR AB ( ((Russian or Spanish or Asian or "Hong Kong" or Mexican) N3 (pandemic* or epidemic*)) ) OR SU ( ((Russian or Spanish or Asian or "Hong Kong" or Mexican) N3 (pandemic* or epidemic*)) )OR KW ( ((Russian or Spanish or Asian or "Hong Kong" or Mexican) N3 (pandemic* or epidemic*)) ) | 72 |
| S5 | TI ((("1889" or 1889-90 or 1889-1890 or 1889-91 or 1889-1891 or 1889-92 or 1889-1892 or 1889-93 or 1889-1893 or 1889-94 or 1889-1894 or 1918-19 or 1918-1919 or 1918-20 or 1918-1920 or "1957" or "1958" or 1957-58 or 1957-1958 or "1968" or 1968-69 or 1968-1969 or 1968-70 or 1968-1970 or "1969" or 1969-70 or 1969-1970 or "1970" or "2009" or 2009-10 or 2009-2010) N6 (pandemic* or epidemic* or outbreak*)) ) OR AB ((("1889" or 1889-90 or 1889-1890 or 1889-91 or 1889-1891 or 1889-92 or 1889-1892 or 1889-93 or 1889-1893 or 1889-94 or 1889-1894 or 1918-19 or 1918-1919 or 1918-20 or 1918-1920 or "1957" or "1958" or 1957-58 or 1957-1958 or "1968" or 1968-69 or 1968-1969 or 1968-70 or 1968-1970 or "1969" or 1969-70 or 1969-1970 or "1970" or "2009" or 2009-10 or 2009-2010) N6 (pandemic* or epidemic* or outbreak*))) OR SU ((("1889" or 1889-90 or 1889-1890 or 1889-91 or 1889-1891 or 1889-92 or 1889-1892 or 1889-93 or 1889-1893 or 1889-94 or 1889-1894 or 1918-19 or 1918-1919 or 1918-20 or 1918-1920 or "1957" or "1958" or 1957-58 or 1957-1958 or "1968" or 1968-69 or 1968-1969 or 1968-70 or 1968-1970 or "1969" or 1969-70 or 1969-1970 or "1970" or "2009" or 2009-10 or 2009-2010) N6 (pandemic* or epidemic* or outbreak*))) OR KW ((("1889" or 1889-90 or 1889-1890 or 1889-91 or 1889-1891 or 1889-92 or 1889-1892 or 1889-93 or 1889-1893 or 1889-94 or 1889-1894 or 1918-19 or 1918-1919 or 1918-20 or 1918-1920 or "1957" or "1958" or 1957-58 or 1957-1958 or "1968" or 1968-69 or 1968-1969 or 1968-70 or 1968-1970 or "1969" or 1969-70 or 1969-1970 or "1970" or "2009" or 2009-10 or 2009-2010) N6(pandemic* or epidemic* or outbreak*))) | 230 |
| S6 | S1 OR S2 OR S3 OR S4 OR S5 | 2,001 |
| S7 | DE "INDIGENOUS peoples of the Americas" OR DE "INDIGENOUS peoples" | 5,952 |
| S8 | DE "NATIVE Americans" OR DE "ESKIMOS" OR DE "FIRST Nations of Canada" OR DE "METIS" OR DE "OFF-reservation Indians (Native Americans)" OR DE "RESERVATION Indians (Native Americans)" | 1,719 |
| S9 | DE "BERBERS -- Ethnic identity" OR DE "INDIGENOUS peoples -- Ethnic identity" OR DE "INDIGENOUS peoples of the Americas -- Ethnic identity" OR DE "MAYAS -- Ethnic identity" OR DE "NATIVE Americans -- Ethnic identity" OR DE "NAVAJO (North American people) -- Ethnic identity" OR DE "CHEROKEE (North American people) -- Ethnic identity" | 135 |
| S10 | DE "MAORI (New Zealand people) -- Ethnic identity" OR DE "PAKEHA (New Zealand people)" OR DE "ARCTIC peoples" OR DE "PACIFIC Islanders" OR DE "METIS" | 403 |
| S11 | TI ( (Indigen* or tribal or tribe* or "first nation*" or native* or nativity or "american indian*" or aborigin*) ) OR AB ( (Indigen* or tribal or tribe* or "first nation*" or native* or nativity or "american indian*" or aborigin*) ) OR SU ( (Indigen* or tribal or tribe* or "first nation*" or native* or nativity or "american indian*" or aborigin*) ) OR KW ( (Indigen* or tribal or tribe* or "first nation*" or native* or nativity or "american indian*" or aborigin*)) | 50,267 |
| S12 | TI (eskimo or eskimos or inuit or inuits or innu or innus or Inupiaq or inuqiaqs or alutiiq or alutiiqs or unangax or unangaxs or yup?ik or yup?iks or cup?ik or cup?iks or athabascan or athabascans or Labrador or Newfoundlander or Newfoundlanders or Newfoundland or “na-dene” or “na-denes” or athabaskan or athabaskans or haida or haidas or tlingit or tlingits or eyak or eyaks or tsimshian or tsimshians or uralic or uralics or samoyed or samoyeds or nenets or nganasan or nganasans or “finno-ugric” or karelian or karelians or khanty or khanties or komi or komis or mansi or mansis or sami or udmurt or udmurts or “tai-kadai” or “tai-kadais” or tai or tais or zhuang or “sino-tibetan” or “sino-tibetans” or “tibeto-burman” or “tibeto-burmans” or bamar or bamars or jingpo or jingpos or karen or karens or meitei or meiteis or naga or newar or tamang or pygmy* or pygmies or papuan or papuans or asmar or paleosiberian or paleosiberians or “chukotko-kamchatkan” or “chukotko-kamchatkans” or chukchi or koryak or “nilo-saharan” or “nilo-saharans” or gur or gurs or igbo or igbos or ijaw or jiaws or mande or mandes or nuba or nubas or senegambian or senegambians or yoruba or yorubas or negrito or negritos or aeta or aetas or andamanese or mamanwa or mamanwas or khoisan or khoisans or khoikhoi or khoikhois or “san people” or “san peoples” or hmong or hmongs or mien or miens or aleut or aleuts or inupiat or inupitats or hadza or hadzas or dravidian or dravidians or kannadiga or kannadigas or malayalam or malayalams or tamil or tamils or telugu or telugus or austronesian or austronesians or atayal or atayals or bunun or bununs or paiwan or paiwans or yami or yamis or “malayo-polynesian” or “malayo-polynesians” or batak or bataks or chamorro or chamorros or malay or malays or maori or maoris or samoan or samoans or austroasiatic or khmer or khmers or kinh or kinhs or nicobarese or “orang asli” or munda or mundas or “torres strait” or “pacific islanders” or “pacific islander” or “pacific people” or “pacific peoples” or tiwi or tiwis or amerind or amerinds or sioux or siouxs or lakota or lakotas or “uto-aztecan” or “uto-aztecans” or huichol or huichols or nahua or nahuas or pima or pimas or tarahumara or tarahumaras or tepehuano or tepehuanos or “tohono o?odham” or “tohono o?odhams” or yaqui or yaquis or penutian or penutians or zuni or zunis or muscogee or muscogees or choctaw or choctaws or seminole or seminoles or “oto-manguean” or “oto-mandueans” or mazahua or mazahuas or mazatec or mazatecs or mixtec or mixtexs or otomi or otomis or zapotec or zapotecs or iroquois or cherokee or cherokees or mohawk or mohawks or caddoan or caddoans or arikara or arikaras or maya or mayas or ojibway orojibways or algonkian or algonkians or algonquin or algonquins or chippewa or chippewas or cree or crees or aymara or aymaras or mapuche or mapuches or quechua or quechuas or waorani or waoranis or arawak or arawaks or “chibchan-paezan” or “chibchan-paezans” or chibcha or chibchas or purepecha or purepechas or warao or waraos or yanomami or yanomamis or jivaro or jivaros or kaingang or kainangs or piaroa or piaroas or ticuna or ticunas or guarani or guaranis or tupi or tupis or mongolic or altaic or buryat or buryats or daur or daurs or kalmyk or kalmyks or mongolian or mongolians or tungusic or tungusics or evenk or evenks or manchu or manchus or oroqen or oroqens or turkic or turkics or azeri or azeris or bashkir or bashkirs or kazakh or kazakhs or kyrgyz or tatar or tatars or uygur or uygurs or yakut or yakuts or “afro-asiatic*” or berber or berbers or tuareg or tuaregs or chadic or chadics or hausa or hausas or cushitic or oromo or oromos or somali or somalis or amhara or amharas or druze or druzes or bedouin or bedouins or gypsy or gypsies or roma or romas) OR AB (eskimo or eskimos or inuit or inuits or innu or innus or Inupiaq or inuqiaqs or alutiiq or alutiiqs or unangax or unangaxs or yup?ik or yup?iks or cup?ik or cup?iks or athabascan or athabascans or Labrador or Newfoundlander or Newfoundlanders or Newfoundland or “na-dene” or “na-denes” or athabaskan or athabaskans or haida or haidas or tlingit or tlingits or eyak or eyaks or tsimshian or tsimshians or uralic or uralics or samoyed or samoyeds or nenets or nganasan or nganasans or “finno-ugric” or karelian or karelians or khanty or khanties or komi or komis or mansi or mansis or sami or udmurt or udmurts or “tai-kadai” or “tai-kadais” or tai or tais or zhuang or “sino-tibetan” or “sino-tibetans” or “tibeto-burman” or “tibeto-burmans” or bamar or bamars or jingpo or jingpos or karen or karens or meitei or meiteis or naga or newar or tamang or pygmy* or pygmies or papuan or papuans or asmar or paleosiberian or paleosiberians or “chukotko-kamchatkan” or “chukotko-kamchatkans” or chukchi or koryak or “nilo-saharan” or “nilo-saharans” or gur or gurs or igbo or igbos or ijaw or jiaws or mande or mandes or nuba or nubas or senegambian or senegambians or yoruba or yorubas or negrito or negritos or aeta or aetas or andamanese or mamanwa or mamanwas or khoisan or khoisans or khoikhoi or khoikhois or “san people” or “san peoples” or hmong or hmongs or mien or miens or aleut or aleuts or inupiat or inupitats or hadza or hadzas or dravidian or dravidians or kannadiga or kannadigas or malayalam or malayalams or tamil or tamils or telugu or telugus or austronesian or austronesians or atayal or atayals or bunun or bununs or paiwan or paiwans or yami or yamis or “malayo-polynesian” or “malayo-polynesians” or batak or bataks or chamorro or chamorros or malay or malays or maori or maoris or samoan or samoans or austroasiatic or khmer or khmers or kinh or kinhs or nicobarese or “orang asli” or munda or mundas or “torres strait” or “pacific islanders” or “pacific islander” or “pacific people” or “pacific peoples” or tiwi or tiwis or amerind or amerinds or sioux or siouxs or lakota or lakotas or “uto-aztecan” or “uto-aztecans” or huichol or huichols or nahua or nahuas or pima or pimas or tarahumara or tarahumaras or tepehuano or tepehuanos or “tohono o?odham” or “tohono o?odhams” or yaqui or yaquis or penutian or penutians or zuni or zunis or muscogee or muscogees or choctaw or choctaws or seminole or seminoles or “oto-manguean” or “oto-mandueans” or mazahua or mazahuas or mazatec or mazatecs or mixtec or mixtexs or otomi or otomis or zapotec or zapotecs or iroquois or cherokee or cherokees or mohawk or mohawks or caddoan or caddoans or arikara or arikaras or maya or mayas or ojibway orojibways or algonkian or algonkians or algonquin or algonquins or chippewa or chippewas or cree or crees or aymara or aymaras or mapuche or mapuches or quechua or quechuas or waorani or waoranis or arawak or arawaks or “chibchan-paezan” or “chibchan-paezans” or chibcha or chibchas or purepecha or purepechas or warao or waraos or yanomami or yanomamis or jivaro or jivaros or kaingang or kainangs or piaroa or piaroas or ticuna or ticunas or guarani or guaranis or tupi or tupis or mongolic or altaic or buryat or buryats or daur or daurs or kalmyk or kalmyks or mongolian or mongolians or tungusic or tungusics or evenk or evenks or manchu or manchus or oroqen or oroqens or turkic or turkics or azeri or azeris or bashkir or bashkirs or kazakh or kazakhs or kyrgyz or tatar or tatars or uygur or uygurs or yakut or yakuts or “afro-asiatic*” or berber or berbers or tuareg or tuaregs or chadic or chadics or hausa or hausas or cushitic or oromo or oromos or somali or somalis or amhara or amharas or druze or druzes or bedouin or bedouins or gypsy or gypsies or roma or romas) OR SU (eskimo or eskimos or inuit or inuits or innu or innus or Inupiaq or inuqiaqs or alutiiq or alutiiqs or unangax or unangaxs or yup?ik or yup?iks or cup?ik or cup?iks or athabascan or athabascans or Labrador or Newfoundlander or Newfoundlanders or Newfoundland or “na-dene” or “na-denes” or athabaskan or athabaskans or haida or haidas or tlingit or tlingits or eyak or eyaks or tsimshian or tsimshians or uralic or uralics or samoyed or samoyeds or nenets or nganasan or nganasans or “finno-ugric” or karelian or karelians or khanty or khanties or komi or komis or mansi or mansis or sami or udmurt or udmurts or “tai-kadai” or “tai-kadais” or tai or tais or zhuang or “sino-tibetan” or “sino-tibetans” or “tibeto-burman” or “tibeto-burmans” or bamar or bamars or jingpo or jingpos or karen or karens or meitei or meiteis or naga or newar or tamang or pygmy* or pygmies or papuan or papuans or asmar or paleosiberian or paleosiberians or “chukotko-kamchatkan” or “chukotko-kamchatkans” or chukchi or koryak or “nilo-saharan” or “nilo-saharans” or gur or gurs or igbo or igbos or ijaw or jiaws or mande or mandes or nuba or nubas or senegambian or senegambians or yoruba or yorubas or negrito or negritos or aeta or aetas or andamanese or mamanwa or mamanwas or khoisan or khoisans or khoikhoi or khoikhois or “san people” or “san peoples” or hmong or hmongs or mien or miens or aleut or aleuts or inupiat or inupitats or hadza or hadzas or dravidian or dravidians or kannadiga or kannadigas or malayalam or malayalams or tamil or tamils or telugu or telugus or austronesian or austronesians or atayal or atayals or bunun or bununs or paiwan or paiwans or yami or yamis or “malayo-polynesian” or “malayo-polynesians” or batak or bataks or chamorro or chamorros or malay or malays or maori or maoris or samoan or samoans or austroasiatic or khmer or khmers or kinh or kinhs or nicobarese or “orang asli” or munda or mundas or “torres strait” or “pacific islanders” or “pacific islander” or “pacific people” or “pacific peoples” or tiwi or tiwis or amerind or amerinds or sioux or siouxs or lakota or lakotas or “uto-aztecan” or “uto-aztecans” or huichol or huichols or nahua or nahuas or pima or pimas or tarahumara or tarahumaras or tepehuano or tepehuanos or “tohono o?odham” or “tohono o?odhams” or yaqui or yaquis or penutian or penutians or zuni or zunis or muscogee or muscogees or choctaw or choctaws or seminole or seminoles or “oto-manguean” or “oto-mandueans” or mazahua or mazahuas or mazatec or mazatecs or mixtec or mixtexs or otomi or otomis or zapotec or zapotecs or iroquois or cherokee or cherokees or mohawk or mohawks or caddoan or caddoans or arikara or arikaras or maya or mayas or ojibway orojibways or algonkian or algonkians or algonquin or algonquins or chippewa or chippewas or cree or crees or aymara or aymaras or mapuche or mapuches or quechua or quechuas or waorani or waoranis or arawak or arawaks or “chibchan-paezan” or “chibchan-paezans” or chibcha or chibchas or purepecha or purepechas or warao or waraos or yanomami or yanomamis or jivaro or jivaros or kaingang or kainangs or piaroa or piaroas or ticuna or ticunas or guarani or guaranis or tupi or tupis or mongolic or altaic or buryat or buryats or daur or daurs or kalmyk or kalmyks or mongolian or mongolians or tungusic or tungusics or evenk or evenks or manchu or manchus or oroqen or oroqens or turkic or turkics or azeri or azeris or bashkir or bashkirs or kazakh or kazakhs or kyrgyz or tatar or tatars or uygur or uygurs or yakut or yakuts or “afro-asiatic*” or berber or berbers or tuareg or tuaregs or chadic or chadics or hausa or hausas or cushitic or oromo or oromos or somali or somalis or amhara or amharas or druze or druzes or bedouin or bedouins or gypsy or gypsies or roma or romas) OR KW (eskimo or eskimos or inuit or inuits or innu or innus or Inupiaq or inuqiaqs or alutiiq or alutiiqs or unangax or unangaxs or yup?ik or yup?iks or cup?ik or cup?iks or athabascan or athabascans or Labrador or Newfoundlander or Newfoundlanders or Newfoundland or “na-dene” or “na-denes” or athabaskan or athabaskans or haida or haidas or tlingit or tlingits or eyak or eyaks or tsimshian or tsimshians or uralic or uralics or samoyed or samoyeds or nenets or nganasan or nganasans or “finno-ugric” or karelian or karelians or khanty or khanties or komi or komis or mansi or mansis or sami or udmurt or udmurts or “tai-kadai” or “tai-kadais” or tai or tais or zhuang or “sino-tibetan” or “sino-tibetans” or “tibeto-burman” or “tibeto-burmans” or bamar or bamars or jingpo or jingpos or karen or karens or meitei or meiteis or naga or newar or tamang or pygmy* or pygmies or papuan or papuans or asmar or paleosiberian or paleosiberians or “chukotko-kamchatkan” or “chukotko-kamchatkans” or chukchi or koryak or “nilo-saharan” or “nilo-saharans” or gur or gurs or igbo or igbos or ijaw or jiaws or mande or mandes or nuba or nubas or senegambian or senegambians or yoruba or yorubas or negrito or negritos or aeta or aetas or andamanese or mamanwa or mamanwas or khoisan or khoisans or khoikhoi or khoikhois or “san people” or “san peoples” or hmong or hmongs or mien or miens or aleut or aleuts or inupiat or inupitats or hadza or hadzas or dravidian or dravidians or kannadiga or kannadigas or malayalam or malayalams or tamil or tamils or telugu or telugus or austronesian or austronesians or atayal or atayals or bunun or bununs or paiwan or paiwans or yami or yamis or “malayo-polynesian” or “malayo-polynesians” or batak or bataks or chamorro or chamorros or malay or malays or maori or maoris or samoan or samoans or austroasiatic or khmer or khmers or kinh or kinhs or nicobarese or “orang asli” or munda or mundas or “torres strait” or “pacific islanders” or “pacific islander” or “pacific people” or “pacific peoples” or tiwi or tiwis or amerind or amerinds or sioux or siouxs or lakota or lakotas or “uto-aztecan” or “uto-aztecans” or huichol or huichols or nahua or nahuas or pima or pimas or tarahumara or tarahumaras or tepehuano or tepehuanos or “tohono o?odham” or “tohono o?odhams” or yaqui or yaquis or penutian or penutians or zuni or zunis or muscogee or muscogees or choctaw or choctaws or seminole or seminoles or “oto-manguean” or “oto-mandueans” or mazahua or mazahuas or mazatec or mazatecs or mixtec or mixtexs or otomi or otomis or zapotec or zapotecs or iroquois or cherokee or cherokees or mohawk or mohawks or caddoan or caddoans or arikara or arikaras or maya or mayas or ojibway orojibways or algonkian or algonkians or algonquin or algonquins or chippewa or chippewas or cree or crees or aymara or aymaras or mapuche or mapuches or quechua or quechuas or waorani or waoranis or arawak or arawaks or “chibchan-paezan” or “chibchan-paezans” or chibcha or chibchas or purepecha or purepechas or warao or waraos or yanomami or yanomamis or jivaro or jivaros or kaingang or kainangs or piaroa or piaroas or ticuna or ticunas or guarani or guaranis or tupi or tupis or mongolic or altaic or buryat or buryats or daur or daurs or kalmyk or kalmyks or mongolian or mongolians or tungusic or tungusics or evenk or evenks or manchu or manchus or oroqen or oroqens or turkic or turkics or azeri or azeris or bashkir or bashkirs or kazakh or kazakhs or kyrgyz or tatar or tatars or uygur or uygurs or yakut or yakuts or “afro-asiatic*” or berber or berbers or tuareg or tuaregs or chadic or chadics or hausa or hausas or cushitic or oromo or oromos or somali or somalis or amhara or amharas or druze or druzes or bedouin or bedouins or gypsy or gypsies or roma or romas)OR KW (eskimo or eskimos or inuit or inuits or innu or innus or Inupiaq or inuqiaqs or alutiiq or alutiiqs or unangax or unangaxs or yup?ik or yup?iks or cup?ik or cup?iks or athabascan or athabascans or Labrador or Newfoundlander or Newfoundlanders or Newfoundland or “na-dene” or “na-denes” or athabaskan or athabascans or haida or haidas or tlingit or tlingits or eyak or eyaks or tsimshian or tsimshians or uralic or uralics or samoyed or samoyeds or nenets or nganasan or nganasans or “finno-ugric” or karelian or karelians or khanty or khanties or komi or komis or mansi or mansis or sami or udmurt or udmurts or “tai-kadai” or “tai-kadais” or tai or tais or zhuang or “sino-tibetan” or “sino-tibetans” or “tibeto-burman” or “tibeto-burmans” or bamar or bamars or jingpo or jingpos or karen or karens or meitei or meiteis or naga or newar or tamang or pygmy* or pygmies or papuan or papuans or asmar or paleosiberian or paleosiberians or “chukotko-kamchatkan” or “chukotko-kamchatkans” or chukchi or koryak or “nilo-saharan” or “nilo-saharans” or gur or gurs or igbo or igbos or ijaw or jiaws or mande or mandes or nuba or nubas or senegambian or senegambians or yoruba or yorubas or negrito or negritos or aeta or aetas or andamanese or mamanwa or mamanwas or khoisan or khoisans or khoikhoi or khoikhois or “san people” or “san peoples” or hmong or hmongs or mien or miens or aleut or aleuts or inupiat or inupitats or hadza or hadzas or dravidian or dravidians or kannadiga or kannadigas or malayalam or malayalams or tamil or tamils or telugu or telugus or austronesian or austronesians or atayal or atayals or bunun or bununs or paiwan or paiwans or yami or yamis or “malayo-polynesian” or “malayo-polynesians” or batak or bataks or chamorro or chamorros or malay or malays or maori or maoris or samoan or samoans or austroasiatic or khmer or khmers or kinh or kinhs or nicobarese or “orang asli” or munda or mundas or “torres strait” or “pacific islanders” or “pacific islander” or “pacific people” or “pacific peoples” or tiwi or tiwis or amerind or amerinds or sioux or siouxs or lakota or lakotas or “uto-aztecan” or “uto-aztecans” or huichol or huichols or nahua or nahuas or pima or pimas or tarahumara or tarahumaras or tepehuano or tepehuanos or “tohono o?odham” or “tohono o?odhams” or yaqui or yaquis or penutian or penutians or zuni or zunis or muscogee or muscogees or choctaw or choctaws or seminole or seminoles or “oto-manguean” or “oto-mandueans” or mazahua or mazahuas or mazatec or mazatecs or mixtec or mixtexs or otomi or otomis or zapotec or zapotecs or iroquois or cherokee or cherokees or mohawk or mohawks or caddoan or caddoans or arikara or arikaras or maya or mayas or ojibway orojibways or algonkian or algonkians or algonquin or algonquins or chippewa or chippewas or cree or crees or aymara or aymaras or mapuche or mapuches or quechua or quechuas or waorani or waoranis or arawak or arawaks or “chibchan-paezan” or “chibchan-paezans” or chibcha or chibchas or purepecha or purepechas or warao or waraos or yanomami or yanomamis or jivaro or jivaros or kaingang or kainangs or piaroa or piaroas or ticuna or ticunas or guarani or guaranis or tupi or tupis or mongolic or altaic or buryat or buryats or daur or daurs or kalmyk or kalmyks or mongolian or mongolians or tungusic or tungusics or evenk or evenks or manchu or manchus or oroqen or oroqens or turkic or turkics or azeri or azeris or bashkir or bashkirs or kazakh or kazakhs or kyrgyz or tatar or tatars or uygur or uygurs or yakut or yakuts or “afro-asiatic*” or berber or berbers or tuareg or tuaregs or chadic or chadics or hausa or hausas or cushitic or oromo or oromos or somali or somalis or amhara or amharas or druze or druzes or bedouin or bedouins or gypsy or gypsies or roma or romas) | 27,032 |
| S13 | S7 OR S8 OR S9 OR S10 OR S11 OR S12 | 72,555 |
| S14 | S6 AND S13 | 80 |

**Database**: Academic Search Ultimate via EBSCOhost

**Date**: 08.02.2021

**Results**: 784

| # | Query | Results |
| --- | --- | --- |
| S1 | DE "INFLUENZA" OR DE "INFLUENZA A virus" OR DE "INFLUENZA A virus, H1N1 subtype" OR DE "INFLUENZA A virus, H3N2 subtype" OR DE "INFLUENZA B virus" OR DE "INFLUENZA Epidemic, 1918-1919" OR DE "INFLUENZAVIRUS A" OR DE "INFLUENZAVIRUS B" OR DE "INFLUENZAVIRUS C" | 23,704 |
| S2 | DE "HONG Kong flu, 1968" | 8 |
| S3 | DE "H1N1 influenza" | 7,473 |
| S4 | DE "SEASONAL influenza" | 1,515 |
| S5 | TI(influenza or influenzas or flu or flus or H1N1 or H1N2 or H2N2 or H3N2 or H3N8) OR AB ((influenza or influenzas or flu or flus or H1N1 or H1N2 or H2N2 or H3N2 or H3N8) OR SU (influenza or influenzas or flu or flus or H1N1 or H1N2 or H2N2 or H3N2 or H3N8) OR KW (influenza or influenzas or flu or flus or H1N1 or H1N2 or H2N2 or H3N2 or H3N8) | 75,938 |
| S6 | TI ( (PH1N1 or H1N1pdm09 or H1N1p) ) OR AB ( (PH1N1 or H1N1pdm09 or H1N1p) ) OR SU (PH1N1 or H1N1pdm09 or H1N1p) ) OR KW ( (PH1N1 or H1N1pdm09 or H1N1p) ) | 656 |
| S7 | TI (((Russian or Spanish or Asian or "Hong Kong" or Mexican) N3 (pandemic* or epidemic*))) OR AB (((Russian or Spanish or Asian or "Hong Kong" or Mexican) N3 (pandemic* or epidemic*))) OR SU (((Russian or Spanish or Asian or "Hong Kong" or Mexican) N3 (pandemic* or epidemic*)))OR KW (((Russian or Spanish or Asian or "Hong Kong" or Mexican) N3 (pandemic* or epidemic*))) | 659 |
| S8 | TI ((("1889" or 1889-90 or 1889-1890 or 1889-91 or 1889-1891 or 1889-92 or 1889-1892 or 1889-93 or 1889-1893 or 1889-94 or 1889-1894 or 1918-19 or 1918-1919 or 1918-20 or 1918-1920 or "1957" or "1958" or 1957-58 or 1957-1958 or "1968" or 1968-69 or 1968-1969 or 1968-70 or 1968-1970 or "1969" or 1969-70 or 1969-1970 or "1970" or "2009" or 2009-10 or 2009-2010) N6 (pandemic* or epidemic* or outbreak*)) ) OR AB ((("1889" or 1889-90 or 1889-1890 or 1889-91 or 1889-1891 or 1889-92 or 1889-1892 or 1889-93 or 1889-1893 or 1889-94 or 1889-1894 or 1918-19 or 1918-1919 or 1918-20 or 1918-1920 or "1957" or "1958" or 1957-58 or 1957-1958 or "1968" or 1968-69 or 1968-1969 or 1968-70 or 1968-1970 or "1969" or 1969-70 or 1969-1970 or "1970" or "2009" or 2009-10 or 2009-2010) N6 (pandemic* or epidemic* or outbreak*))) OR SU ((("1889" or 1889-90 or 1889-1890 or 1889-91 or 1889-1891 or 1889-92 or 1889-1892 or 1889-93 or 1889-1893 or 1889-94 or 1889-1894 or 1918-19 or 1918-1919 or 1918-20 or 1918-1920 or "1957" or "1958" or 1957-58 or 1957-1958 or "1968" or 1968-69 or 1968-1969 or 1968-70 or 1968-1970 or "1969" or 1969-70 or 1969-1970 or "1970" or "2009" or 2009-10 or 2009-2010) N7 (pandemic* or epidemic* or outbreak*))) OR KW ((("1889" or 1889-90 or 1889-1890 or 1889-91 or 1889-1891 or 1889-92 or 1889-1892 or 1889-93 or 1889-1893 or 1889-94 or 1889-1894 or 1918-19 or 1918-1919 or 1918-20 or 1918-1920 or "1957" or "1958" or 1957-58 or 1957-1958 or "1968" or 1968-69 or 1968-1969 or 1968-70 or 1968-1970 or "1969" or 1969-70 or 1969-1970 or "1970" or "2009" or 2009-10 or 2009-2010) N6 (pandemic* or epidemic* or outbreak*))) | 5,952 |
| S9 | S1 OR S2 OR S3 OR S4 OR S5 OR S6 OR S7 OR S8 | 76,874 |
| S10 | SU ("american people*" or "african people*" or "asian people*" or "european people" or "australian people*" or "polynesian people*" or "micronesian people*" or "New Guinean people*" or "pacific people*" or "arctic people*" or "new zealand people*") | 23,669 |
| S11 | DE "NATIVE Americans" DE "INUIT" OR DE "ESKIMOS" OR DE "ALASKA Natives" OR DE "ALEUTS" OR DE "FIRST Nations of Canada" OR DE "ARCTIC peoples" OR DE "SKOLTS (Sami people)" | 2,846 |
| S12 | DE "INDIGENOUS peoples of the Americas" OR DE "INDIGENOUS peoples of Central America" OR DE "INDIGENOUS peoples of Mexico" OR DE "INDIGENOUS peoples of South America" OR DE "INDIGENOUS peoples of the West Indies" OR DE "INDIGENOUS peoples -- Hispaniola" OR DE "INDIGENOUS peoples -- Amazon River Valley" | 5,565 |
| S13 | DE "INDIGENOUS Australians" OR DE "ABORIGINAL Australians" OR DE "ABORIGINAL Tasmanians" OR DE "TORRES Strait Islanders" | 4,861 |
| S14 | DE "TAIWAN aborigines" | 77 |
| S15 | TI ( (Indigen* or tribal or tribe* or "first nation*" or native* or nativity or "american indian*" or aborigin*) ) OR AB ( (Indigen* or tribal or tribe* or "first nation*" or native* or nativity or "american indian*" or aborigin*) ) OR SU ( (Indigen* or tribal or tribe* or "first nation*" or native* or nativity or "american indian*" or aborigin*) ) OR KW ( (Indigen* or tribal or tribe* or "first nation*" or native* or nativity or "american indian*" or aborigin*)) | 358,159 |
| S16 | TI (eskimo or eskimos or inuit or inuits or innu or innus or Inupiaq or inuqiaqs or alutiiq or alutiiqs or unangax or unangaxs or yup?ik or yup?iks or cup?ik or cup?iks or athabascan or athabascans or Labrador or Newfoundlander or Newfoundlanders or Newfoundland or “na-dene” or “na-denes” or athabaskan or athabaskans or haida or haidas or tlingit or tlingits or eyak or eyaks or tsimshian or tsimshians or uralic or uralics or samoyed or samoyeds or nenets or nganasan or nganasans or “finno-ugric” or karelian or karelians or khanty or khanties or komi or komis or mansi or mansis or sami or udmurt or udmurts or “tai-kadai” or “tai-kadais” or tai or tais or zhuang or “sino-tibetan” or “sino-tibetans” or “tibeto-burman” or “tibeto-burmans” or bamar or bamars or jingpo or jingpos or karen or karens or meitei or meiteis or naga or newar or tamang or pygmy* or pygmies or papuan or papuans or asmar or paleosiberian or paleosiberians or “chukotko-kamchatkan” or “chukotko-kamchatkans” or chukchi or koryak or “nilo-saharan” or “nilo-saharans” or gur or gurs or igbo or igbos or ijaw or jiaws or mande or mandes or nuba or nubas or senegambian or senegambians or yoruba or yorubas or negrito or negritos or aeta or aetas or andamanese or mamanwa or mamanwas or khoisan or khoisans or khoikhoi or khoikhois or “san people” or “san peoples” or hmong or hmongs or mien or miens or aleut or aleuts or inupiat or inupitats or hadza or hadzas or dravidian or dravidians or kannadiga or kannadigas or malayalam or malayalams or tamil or tamils or telugu or telugus or austronesian or austronesians or atayal or atayals or bunun or bununs or paiwan or paiwans or yami or yamis or “malayo-polynesian” or “malayo-polynesians” or batak or bataks or chamorro or chamorros or malay or malays or maori or maoris or samoan or samoans or austroasiatic or khmer or khmers or kinh or kinhs or nicobarese or “orang asli” or munda or mundas or “torres strait” or “pacific islanders” or “pacific islander” or “pacific people” or “pacific peoples” or tiwi or tiwis or amerind or amerinds or sioux or siouxs or lakota or lakotas or “uto-aztecan” or “uto-aztecans” or huichol or huichols or nahua or nahuas or pima or pimas or tarahumara or tarahumaras or tepehuano or tepehuanos or “tohono o?odham” or “tohono o?odhams” or yaqui or yaquis or penutian or penutians or zuni or zunis or muscogee or muscogees or choctaw or choctaws or seminole or seminoles or “oto-manguean” or “oto-mandueans” or mazahua or mazahuas or mazatec or mazatecs or mixtec or mixtexs or otomi or otomis or zapotec or zapotecs or iroquois or cherokee or cherokees or mohawk or mohawks or caddoan or caddoans or arikara or arikaras or maya or mayas or ojibway orojibways or algonkian or algonkians or algonquin or algonquins or chippewa or chippewas or cree or crees or aymara or aymaras or mapuche or mapuches or quechua or quechuas or waorani or waoranis or arawak or arawaks or “chibchan-paezan” or “chibchan-paezans” or chibcha or chibchas or purepecha or purepechas or warao or waraos or yanomami or yanomamis or jivaro or jivaros or kaingang or kainangs or piaroa or piaroas or ticuna or ticunas or guarani or guaranis or tupi or tupis or mongolic or altaic or buryat or buryats or daur or daurs or kalmyk or kalmyks or mongolian or mongolians or tungusic or tungusics or evenk or evenks or manchu or manchus or oroqen or oroqens or turkic or turkics or azeri or azeris or bashkir or bashkirs or kazakh or kazakhs or kyrgyz or tatar or tatars or uygur or uygurs or yakut or yakuts or “afro-asiatic*” or berber or berbers or tuareg or tuaregs or chadic or chadics or hausa or hausas or cushitic or oromo or oromos or somali or somalis or amhara or amharas or druze or druzes or bedouin or bedouins or gypsy or gypsies or roma or romas) OR AB (eskimo or eskimos or inuit or inuits or innu or innus or Inupiaq or inuqiaqs or alutiiq or alutiiqs or unangax or unangaxs or yup?ik or yup?iks or cup?ik or cup?iks or athabascan or athabascans or Labrador or Newfoundlander or Newfoundlanders or Newfoundland or “na-dene” or “na-denes” or athabaskan or athabaskans or haida or haidas or tlingit or tlingits or eyak or eyaks or tsimshian or tsimshians or uralic or uralics or samoyed or samoyeds or nenets or nganasan or nganasans or “finno-ugric” or karelian or karelians or khanty or khanties or komi or komis or mansi or mansis or sami or udmurt or udmurts or “tai-kadai” or “tai-kadais” or tai or tais or zhuang or “sino-tibetan” or “sino-tibetans” or “tibeto-burman” or “tibeto-burmans” or bamar or bamars or jingpo or jingpos or karen or karens or meitei or meiteis or naga or newar or tamang or pygmy* or pygmies or papuan or papuans or asmar or paleosiberian or paleosiberians or “chukotko-kamchatkan” or “chukotko-kamchatkans” or chukchi or koryak or “nilo-saharan” or “nilo-saharans” or gur or gurs or igbo or igbos or ijaw or jiaws or mande or mandes or nuba or nubas or senegambian or senegambians or yoruba or yorubas or negrito or negritos or aeta or aetas or andamanese or mamanwa or mamanwas or khoisan or khoisans or khoikhoi or khoikhois or “san people” or “san peoples” or hmong or hmongs or mien or miens or aleut or aleuts or inupiat or inupitats or hadza or hadzas or dravidian or dravidians or kannadiga or kannadigas or malayalam or malayalams or tamil or tamils or telugu or telugus or austronesian or austronesians or atayal or atayals or bunun or bununs or paiwan or paiwans or yami or yamis or “malayo-polynesian” or “malayo-polynesians” or batak or bataks or chamorro or chamorros or malay or malays or maori or maoris or samoan or samoans or austroasiatic or khmer or khmers or kinh or kinhs or nicobarese or “orang asli” or munda or mundas or “torres strait” or “pacific islanders” or “pacific islander” or “pacific people” or “pacific peoples” or tiwi or tiwis or amerind or amerinds or sioux or siouxs or lakota or lakotas or “uto-aztecan” or “uto-aztecans” or huichol or huichols or nahua or nahuas or pima or pimas or tarahumara or tarahumaras or tepehuano or tepehuanos or “tohono o?odham” or “tohono o?odhams” or yaqui or yaquis or penutian or penutians or zuni or zunis or muscogee or muscogees or choctaw or choctaws or seminole or seminoles or “oto-manguean” or “oto-mandueans” or mazahua or mazahuas or mazatec or mazatecs or mixtec or mixtexs or otomi or otomis or zapotec or zapotecs or iroquois or cherokee or cherokees or mohawk or mohawks or caddoan or caddoans or arikara or arikaras or maya or mayas or ojibway orojibways or algonkian or algonkians or algonquin or algonquins or chippewa or chippewas or cree or crees or aymara or aymaras or mapuche or mapuches or quechua or quechuas or waorani or waoranis or arawak or arawaks or “chibchan-paezan” or “chibchan-paezans” or chibcha or chibchas or purepecha or purepechas or warao or waraos or yanomami or yanomamis or jivaro or jivaros or kaingang or kainangs or piaroa or piaroas or ticuna or ticunas or guarani or guaranis or tupi or tupis or mongolic or altaic or buryat or buryats or daur or daurs or kalmyk or kalmyks or mongolian or mongolians or tungusic or tungusics or evenk or evenks or manchu or manchus or oroqen or oroqens or turkic or turkics or azeri or azeris or bashkir or bashkirs or kazakh or kazakhs or kyrgyz or tatar or tatars or uygur or uygurs or yakut or yakuts or “afro-asiatic*” or berber or berbers or tuareg or tuaregs or chadic or chadics or hausa or hausas or cushitic or oromo or oromos or somali or somalis or amhara or amharas or druze or druzes or bedouin or bedouins or gypsy or gypsies or roma or romas) OR SU (eskimo or eskimos or inuit or inuits or innu or innus or Inupiaq or inuqiaqs or alutiiq or alutiiqs or unangax or unangaxs or yup?ik or yup?iks or cup?ik or cup?iks or athabascan or athabascans or Labrador or Newfoundlander or Newfoundlanders or Newfoundland or “na-dene” or “na-denes” or athabaskan or athabaskans or haida or haidas or tlingit or tlingits or eyak or eyaks or tsimshian or tsimshians or uralic or uralics or samoyed or samoyeds or nenets or nganasan or nganasans or “finno-ugric” or karelian or karelians or khanty or khanties or komi or komis or mansi or mansis or sami or udmurt or udmurts or “tai-kadai” or “tai-kadais” or tai or tais or zhuang or “sino-tibetan” or “sino-tibetans” or “tibeto-burman” or “tibeto-burmans” or bamar or bamars or jingpo or jingpos or karen or karens or meitei or meiteis or naga or newar or tamang or pygmy* or pygmies or papuan or papuans or asmar or paleosiberian or paleosiberians or “chukotko-kamchatkan” or “chukotko-kamchatkans” or chukchi or koryak or “nilo-saharan” or “nilo-saharans” or gur or gurs or igbo or igbos or ijaw or jiaws or mande or mandes or nuba or nubas or senegambian or senegambians or yoruba or yorubas or negrito or negritos or aeta or aetas or andamanese or mamanwa or mamanwas or khoisan or khoisans or khoikhoi or khoikhois or “san people” or “san peoples” or hmong or hmongs or mien or miens or aleut or aleuts or inupiat or inupitats or hadza or hadzas or dravidian or dravidians or kannadiga or kannadigas or malayalam or malayalams or tamil or tamils or telugu or telugus or austronesian or austronesians or atayal or atayals or bunun or bununs or paiwan or paiwans or yami or yamis or “malayo-polynesian” or “malayo-polynesians” or batak or bataks or chamorro or chamorros or malay or malays or maori or maoris or samoan or samoans or austroasiatic or khmer or khmers or kinh or kinhs or nicobarese or “orang asli” or munda or mundas or “torres strait” or “pacific islanders” or “pacific islander” or “pacific people” or “pacific peoples” or tiwi or tiwis or amerind or amerinds or sioux or siouxs or lakota or lakotas or “uto-aztecan” or “uto-aztecans” or huichol or huichols or nahua or nahuas or pima or pimas or tarahumara or tarahumaras or tepehuano or tepehuanos or “tohono o?odham” or “tohono o?odhams” or yaqui or yaquis or penutian or penutians or zuni or zunis or muscogee or muscogees or choctaw or choctaws or seminole or seminoles or “oto-manguean” or “oto-mandueans” or mazahua or mazahuas or mazatec or mazatecs or mixtec or mixtexs or otomi or otomis or zapotec or zapotecs or iroquois or cherokee or cherokees or mohawk or mohawks or caddoan or caddoans or arikara or arikaras or maya or mayas or ojibway orojibways or algonkian or algonkians or algonquin or algonquins or chippewa or chippewas or cree or crees or aymara or aymaras or mapuche or mapuches or quechua or quechuas or waorani or waoranis or arawak or arawaks or “chibchan-paezan” or “chibchan-paezans” or chibcha or chibchas or purepecha or purepechas or warao or waraos or yanomami or yanomamis or jivaro or jivaros or kaingang or kainangs or piaroa or piaroas or ticuna or ticunas or guarani or guaranis or tupi or tupis or mongolic or altaic or buryat or buryats or daur or daurs or kalmyk or kalmyks or mongolian or mongolians or tungusic or tungusics or evenk or evenks or manchu or manchus or oroqen or oroqens or turkic or turkics or azeri or azeris or bashkir or bashkirs or kazakh or kazakhs or kyrgyz or tatar or tatars or uygur or uygurs or yakut or yakuts or “afro-asiatic*” or berber or berbers or tuareg or tuaregs or chadic or chadics or hausa or hausas or cushitic or oromo or oromos or somali or somalis or amhara or amharas or druze or druzes or bedouin or bedouins or gypsy or gypsies or roma or romas) OR KW (eskimo or eskimos or inuit or inuits or innu or innus or Inupiaq or inuqiaqs or alutiiq or alutiiqs or unangax or unangaxs or yup?ik or yup?iks or cup?ik or cup?iks or athabascan or athabascans or Labrador or Newfoundlander or Newfoundlanders or Newfoundland or “na-dene” or “na-denes” or athabaskan or athabaskans or haida or haidas or tlingit or tlingits or eyak or eyaks or tsimshian or tsimshians or uralic or uralics or samoyed or samoyeds or nenets or nganasan or nganasans or “finno-ugric” or karelian or karelians or khanty or khanties or komi or komis or mansi or mansis or sami or udmurt or udmurts or “tai-kadai” or “tai-kadais” or tai or tais or zhuang or “sino-tibetan” or “sino-tibetans” or “tibeto-burman” or “tibeto-burmans” or bamar or bamars or jingpo or jingpos or karen or karens or meitei or meiteis or naga or newar or tamang or pygmy* or pygmies or papuan or papuans or asmar or paleosiberian or paleosiberians or “chukotko-kamchatkan” or “chukotko-kamchatkans” or chukchi or koryak or “nilo-saharan” or “nilo-saharans” or gur or gurs or igbo or igbos or ijaw or jiaws or mande or mandes or nuba or nubas or senegambian or senegambians or yoruba or yorubas or negrito or negritos or aeta or aetas or andamanese or mamanwa or mamanwas or khoisan or khoisans or khoikhoi or khoikhois or “san people” or “san peoples” or hmong or hmongs or mien or miens or aleut or aleuts or inupiat or inupitats or hadza or hadzas or dravidian or dravidians or kannadiga or kannadigas or malayalam or malayalams or tamil or tamils or telugu or telugus or austronesian or austronesians or atayal or atayals or bunun or bununs or paiwan or paiwans or yami or yamis or “malayo-polynesian” or “malayo-polynesians” or batak or bataks or chamorro or chamorros or malay or malays or maori or maoris or samoan or samoans or austroasiatic or khmer or khmers or kinh or kinhs or nicobarese or “orang asli” or munda or mundas or “torres strait” or “pacific islanders” or “pacific islander” or “pacific people” or “pacific peoples” or tiwi or tiwis or amerind or amerinds or sioux or siouxs or lakota or lakotas or “uto-aztecan” or “uto-aztecans” or huichol or huichols or nahua or nahuas or pima or pimas or tarahumara or tarahumaras or tepehuano or tepehuanos or “tohono o?odham” or “tohono o?odhams” or yaqui or yaquis or penutian or penutians or zuni or zunis or muscogee or muscogees or choctaw or choctaws or seminole or seminoles or “oto-manguean” or “oto-mandueans” or mazahua or mazahuas or mazatec or mazatecs or mixtec or mixtexs or otomi or otomis or zapotec or zapotecs or iroquois or cherokee or cherokees or mohawk or mohawks or caddoan or caddoans or arikara or arikaras or maya or mayas or ojibway orojibways or algonkian or algonkians or algonquin or algonquins or chippewa or chippewas or cree or crees or aymara or aymaras or mapuche or mapuches or quechua or quechuas or waorani or waoranis or arawak or arawaks or “chibchan-paezan” or “chibchan-paezans” or chibcha or chibchas or purepecha or purepechas or warao or waraos or yanomami or yanomamis or jivaro or jivaros or kaingang or kainangs or piaroa or piaroas or ticuna or ticunas or guarani or guaranis or tupi or tupis or mongolic or altaic or buryat or buryats or daur or daurs or kalmyk or kalmyks or mongolian or mongolians or tungusic or tungusics or evenk or evenks or manchu or manchus or oroqen or oroqens or turkic or turkics or azeri or azeris or bashkir or bashkirs or kazakh or kazakhs or kyrgyz or tatar or tatars or uygur or uygurs or yakut or yakuts or “afro-asiatic*” or berber or berbers or tuareg or tuaregs or chadic or chadics or hausa or hausas or cushitic or oromo or oromos or somali or somalis or amhara or amharas or druze or druzes or bedouin or bedouins or gypsy or gypsies or roma or romas)OR KW (eskimo or eskimos or inuit or inuits or innu or innus or Inupiaq or inuqiaqs or alutiiq or alutiiqs or unangax or unangaxs or yup?ik or yup?iks or cup?ik or cup?iks or athabascan or athabascans or Labrador or Newfoundlander or Newfoundlanders or Newfoundland or “na-dene” or “na-denes” or athabaskan or athabascans or haida or haidas or tlingit or tlingits or eyak or eyaks or tsimshian or tsimshians or uralic or uralics or samoyed or samoyeds or nenets or nganasan or nganasans or “finno-ugric” or karelian or karelians or khanty or khanties or komi or komis or mansi or mansis or sami or udmurt or udmurts or “tai-kadai” or “tai-kadais” or tai or tais or zhuang or “sino-tibetan” or “sino-tibetans” or “tibeto-burman” or “tibeto-burmans” or bamar or bamars or jingpo or jingpos or karen or karens or meitei or meiteis or naga or newar or tamang or pygmy* or pygmies or papuan or papuans or asmar or paleosiberian or paleosiberians or “chukotko-kamchatkan” or “chukotko-kamchatkans” or chukchi or koryak or “nilo-saharan” or “nilo-saharans” or gur or gurs or igbo or igbos or ijaw or jiaws or mande or mandes or nuba or nubas or senegambian or senegambians or yoruba or yorubas or negrito or negritos or aeta or aetas or andamanese or mamanwa or mamanwas or khoisan or khoisans or khoikhoi or khoikhois or “san people” or “san peoples” or hmong or hmongs or mien or miens or aleut or aleuts or inupiat or inupitats or hadza or hadzas or dravidian or dravidians or kannadiga or kannadigas or malayalam or malayalams or tamil or tamils or telugu or telugus or austronesian or austronesians or atayal or atayals or bunun or bununs or paiwan or paiwans or yami or yamis or “malayo-polynesian” or “malayo-polynesians” or batak or bataks or chamorro or chamorros or malay or malays or maori or maoris or samoan or samoans or austroasiatic or khmer or khmers or kinh or kinhs or nicobarese or “orang asli” or munda or mundas or “torres strait” or “pacific islanders” or “pacific islander” or “pacific people” or “pacific peoples” or tiwi or tiwis or amerind or amerinds or sioux or siouxs or lakota or lakotas or “uto-aztecan” or “uto-aztecans” or huichol or huichols or nahua or nahuas or pima or pimas or tarahumara or tarahumaras or tepehuano or tepehuanos or “tohono o?odham” or “tohono o?odhams” or yaqui or yaquis or penutian or penutians or zuni or zunis or muscogee or muscogees or choctaw or choctaws or seminole or seminoles or “oto-manguean” or “oto-mandueans” or mazahua or mazahuas or mazatec or mazatecs or mixtec or mixtexs or otomi or otomis or zapotec or zapotecs or iroquois or cherokee or cherokees or mohawk or mohawks or caddoan or caddoans or arikara or arikaras or maya or mayas or ojibway orojibways or algonkian or algonkians or algonquin or algonquins or chippewa or chippewas or cree or crees or aymara or aymaras or mapuche or mapuches or quechua or quechuas or waorani or waoranis or arawak or arawaks or “chibchan-paezan” or “chibchan-paezans” or chibcha or chibchas or purepecha or purepechas or warao or waraos or yanomami or yanomamis or jivaro or jivaros or kaingang or kainangs or piaroa or piaroas or ticuna or ticunas or guarani or guaranis or tupi or tupis or mongolic or altaic or buryat or buryats or daur or daurs or kalmyk or kalmyks or mongolian or mongolians or tungusic or tungusics or evenk or evenks or manchu or manchus or oroqen or oroqens or turkic or turkics or azeri or azeris or bashkir or bashkirs or kazakh or kazakhs or kyrgyz or tatar or tatars or uygur or uygurs or yakut or yakuts or “afro-asiatic*” or berber or berbers or tuareg or tuaregs or chadic or chadics or hausa or hausas or cushitic or oromo or oromos or somali or somalis or amhara or amharas or druze or druzes or bedouin or bedouins or gypsy or gypsies or roma or romas) | 175,240 |
| S17 | S10 OR S11 OR S12 OR S13 OR S14 OR S15 OR S16 | 522,214 |
| S18 | S9 AND S17 | 886 |
| S19 | S9 AND S17 scholarly journals | 784 |

**Database**: ASSIA via ProQuest

**Date**: 09.02.2021

**Results**: 107

(MAINSUBJECT.EXACT("Influenza") OR NOFT(Influenza or influenzas or influenzavirus or flu or flus or H1N1 or H1N2 or H2N2 or H3N2 or H3N8) OR NOFT(PH1N1 or H1N1pdm09 or H1N1p) OR NOFT((Russian or Spanish or Asian or "Hong Kong" or Mexican) NEAR/3 (pandemic* or epidemic*)) OR NOFT(("1889" or 1889-90 or 1889-1890 or 1889-91 or 1889-1891 or 1889-92 or 1889-1892 or 1889-93 or 1889-1893 or 1889-94 or 1889-1894 or 1918-19 or 1918-1919 or 1918-20 or 1918-1920 or "1957" or "1958" or 1957-58 or 1957-1958 or "1968" or 1968-69 or 1968-1969 or 1968-70 or 1968-1970 or "1969" or 1969-70 or 1969-1970 or "1970" or "2009" or 2009-10 or 2009-2010) NEAR/6 (pandemic* or epidemic* or outbreak*))) AND ((MAINSUBJECT.EXACT.EXPLODE("Indigenous people") OR MAINSUBJECT.EXACT.EXPLODE("Primitive societies") OR MAINSUBJECT.EXACT.EXPLODE("Pacific Islander people") OR MAINSUBJECT.EXACT.EXPLODE("Papua New Guinean people") OR MAINSUBJECT.EXACT.EXPLODE("Eskimos") OR MAINSUBJECT.EXACT("Zongo communities") OR MAINSUBJECT.EXACT("Romany communities") OR MAINSUBJECT.EXACT("Afro-Caribbean communities") OR MAINSUBJECT.EXACT("Asian communities") OR MAINSUBJECT.EXACT("West Indian communities") OR MAINSUBJECT.EXACT("American Samoan communities") OR MAINSUBJECT.EXACT ("American Indian communities") OR MAINSUBJECT.EXACT("Yemeni communities") OR MAINSUBJECT.EXACT("African communities") OR MAINSUBJECT.EXACT("Nubian people") OR MAINSUBJECT.EXACT("Yoruba people") OR MAINSUBJECT.EXACT("Igbo people") OR MAINSUBJECT.EXACT("Tver' Karelian people") OR MAINSUBJECT.EXACT("Dinka people") OR MAINSUBJECT.EXACT("Kalenjin people") OR MAINSUBJECT.EXACT("Manyika people") OR MAINSUBJECT.EXACT.EXPLODE("Polynesian people") OR MAINSUBJECT.EXACT("Toba Batak people") OR MAINSUBJECT.EXACT("Chukotka people") OR MAINSUBJECT.EXACT("Nilotic people") OR MAINSUBJECT.EXACT("Khoikhoi people") OR MAINSUBJECT.EXACT("Moluccan people") OR MAINSUBJECT.EXACT("Tamang people") OR MAINSUBJECT.EXACT("Maasai people") OR MAINSUBJECT.EXACT("Ngaju Dayak people") OR MAINSUBJECT.EXACT("Akan people") OR MAINSUBJECT.EXACT("Bantu people") OR MAINSUBJECT.EXACT("Bambara people") OR MAINSUBJECT.EXACT("Minangkabau people") OR MAINSUBJECT.EXACT("Esan people") OR MAINSUBJECT.EXACT("Malaysian people") OR MAINSUBJECT.EXACT("Sri Lankan Tamil people") OR MAINSUBJECT.EXACT("Hmong people") OR MAINSUBJECT.EXACT("Tamil people") OR MAINSUBJECT.EXACT("Polynesian people") OR MAINSUBJECT.EXACT("Semai people") OR MAINSUBJECT.EXACT("Mayawo people") OR MAINSUBJECT.EXACT("Tamil communities") OR MAINSUBJECT.EXACT("Samoan people") OR MAINSUBJECT.EXACT("Kazakh people") OR MAINSUBJECT.EXACT("Oromo people") OR MAINSUBJECT.EXACT("Druze people") OR MAINSUBJECT.EXACT("Turkic people") OR MAINSUBJECT.EXACT("Somali people") OR MAINSUBJECT.EXACT.EXPLODE("Tartar people") OR MAINSUBJECT.EXACT("Bedouin people") OR MAINSUBJECT.EXACT("Caribbean people")) OR NOFT(Indigen* or tribal* or tribe* or "first nation*" or native* or nativity* or "american indian*" or aborigin*) OR NOFT(eskimo* or inuit* or innu* or Inupiaq* or inuqiaq* or alutiiq* or unangax* or yup?ik* or cup?ik* or athabascan* or Labrador* or Newfoundland* or "na-dene" or "na-denes" or athabaskan* or haida* or tlingit* or eyak* or tsimshian* or uralic* or samoyed* or nenet* or nganasan* or "finno-ugric" or karelian* or khanty* or khanties* or komi* or mansi* or sami* or udmurt* or "tai-kadai" or "tai-kadais" or tai or tais or zhuang* or "sino-tibetan" or "sino-tibetans" or "tibeto-burman" or "tibeto-burmans" or bamar* or jingpo* or karen* or meitei* or naga* or newar* or tamang* or pygmy* or pygmies* or Papuan* or asmar* or paleosiberian* or "chukotko-kamchatkan" or "chukotko-kamchatkans" or chukchi* or koryak* or "nilo-saharan" or "nilo-saharans" or gur or gurs or igbo* or igbos* or ijaw* or jiaw* or mande* or nuba* or senegambian* or yoruba* or negrito* or aeta* or andamanese* or mamanwa* or khoisan* or khoikhoi* or "san people" or hmong* or mien* or aleut* or inupiat* or hadza* or dravidian* or kannadiga* or malayalam* or tamil* or telugu* or austronesian* or atayal* or bunun* or paiwan* or yami* or "malayo-polynesian" or "malayo-polynesians" or batak* or chamorro* or malay* or maori* or samoan* or austroasiatic* or khmer* or kinh* or nicobarese* or "orang asli" or munda* or "torres strait" or "pacific islanders" or "pacific islander" or "pacific people" or "pacific peoples" or tiwi* or amerind* or sioux* or lakota* or "uto-aztecan" or "uto-aztecans" or huichol* or nahua* or pima* or tarahumara* or tepehuano* or "tohono o?odham" or "tohono o?odhams" or yaqui* or penutian* or zuni* or muscogee* or choctaw* or seminole* or "oto-manguean" or "oto-mandueans" or mazahua* or mazatec* or mixtec* or otomi* or zapotec* or iroquoi* or cherokee* or mohawk* or caddoan* or arikara* or maya* or ojibway* or algonkian* or algonquin* or chippewa* or cree* or aymara* or mapuche* or quechua* or waorani* or arawak* or "chibchan-paezan" or "chibchan-paezans" or chibcha* or purepecha* or warao* or yanomami* or jivaro* or kaingang* or piaroa* or ticuna* or guarani* or tupi* or mongolic* or altaic* or buryat* or daur* or kalmyk* or mongolian* or tungusic* or evenk* or manchu* or oroqen* or turkic* or azeri* or bashkir* or kazakh* or kyrgyz* or tatar* or uygur* or yakut* or "afro-asiatic" or berber* or tuareg* or chadic* or hausa* or cushitic* or oromo* or somali* or amhara* or druze* or bedouin* or gypsy* or gypsies* or roma*))

**Database**: Google Scholar – via Publish or Perish

**Date**: 11.02.2021

**Results**: 200 første treffene med Google scholars egen relevansrangering

"influenza|flu|H1N1|H1N2|H2N2|H3N2|H3N8" "Indigenous|tribal|tribe|"first nation"|native|nativity|"american indian"|aboriginal|aboriginee"
